# The colour of an avifauna: A quantitative analysis of the colour of Australian birds

**DOI:** 10.1038/srep18514

**Published:** 2015-12-18

**Authors:** Kaspar Delhey

**Affiliations:** 1 School of Biological Sciences, Monash University, Clayton, Victoria, Australia; 2 Max Planck Institute for Ornithology, Radolfzell, 78315, Germany

## Abstract

Animal coloration is a poorly-understood aspect of phenotypic variability. Here I expand initial studies of the colour gamut of birds by providing the first quantitative description of the colour variation of an entire avifauna: Australian landbirds (555 species). The colour of Australian birds occupies a small fraction (19%) of the entire possible colour space and colour variation is extremely uneven. Most colours are unsaturated, concentrated in the centre of colour space and based on the deposition of melanins. Other mechanisms of colour production are less common but account for larger portions of colour space and for most saturated colours. Male colours occupy 45–25% more colour space than female colours, indicating that sexual dichromatism translates into a broader range of male colours. Male-exclusive colours are often saturated, at the edge of chromatic space, and have most likely evolved for signalling. While most clades of birds occupy expected or lower-than-expected colour volumes, parrots and cockatoos (Order Psittaciformes) occupy a much larger volume than expected. This uneven distribution of colour variation across mechanisms of colour production, sexes and clades is probably shared by avifaunas in other parts of the world, but this remains to be tested with comparable data.

Animal colours have fascinated humans for a long time^1^. This long history is reflected in part by the multiple hypotheses put forward to make sense of animal colour diversity. Some of these hypotheses concern non-visual functions of animal colours. For example colours may help with thermoregulation, shield against harmful ultraviolet radiation, protect from parasites and abrasion or reduce glare (see reviews in^2^,^3^). Alternatively, colours may be shaped by their visual functions, such as preventing detection by predators or functioning as advertisement to attract potential mates or deter rivals or predators^4^,^5^. Colours may also be simply by-products of other physiological processes^6^. Despite this multiplicity of explanations it has been difficult to determine their relative importance as general explanations of colour diversity in nature.

Our inability to understand colour diversity could be due to a lack of knowledge of the extent and characteristics of colour variation in nature. Which colours are more common? What mechanisms of colour production account for most colour variation? Do males have more colours than females? Are there certain taxonomic groups that are more colourful than others? Intuitively, many of us would have a tentative answer to most of these questions, but we would hesitate if asked to estimate the magnitude of these effects. That such quantitative assessments are almost entirely lacking may be due to the fact that chromatic variation is difficult to study because of its highly multidimensional nature^7^. Each colour has multiple dimensions (chromatic coordinates, hue, saturation, etc.) and very often species have multiple colour patches over their bodies. Some of these limitations have been overcome by analysing colours using models of colour vision tailored to the appropriate receivers^7^.

Recently, colour variation in birds and plants has been successfully explored using a colour morphospace approach based on models of avian colour vision^8^,^9^. Such studies use current knowledge on avian colour vision to plot colours in visual space (which in birds takes the form of a three-dimensional tetrahedron, Fig. 1) to assess patterns of chromatic variation. These studies revealed that while plumage colours occupy a larger volume in the visual space of birds compared to plant colours, large parts (>70%) of the theoretically possible chromatic space were unoccupied. Furthermore, bird colours tend to occupy different parts of the visual space than plant colours, most likely due to differences in the mechanisms of colour production. Within birds, different mechanisms of colour production occupy highly divergent amounts of the total colour gamut of birds. For example, structural colours, colours produced through physical scattering of light by the microstructure of the feather, account for much larger fractions of the total occupied volume than pigmentary colours^8^.

Initial efforts have provided unique insights into the potential constraints that shape colour evolution in birds^8^. However, given that their aim was to study the total gamut of bird colours, species were selected to represent all known mechanisms of colour production^8^ rather than to represent a sample of birds as a whole, and only males were sampled. Thus, sampling may be biased towards flashy or conspicuous colours, which capture extreme variation but do not represent a random sample. While the only way to eliminate sampling bias completely would be to measure all bird species in the world, a representative sample can be obtained by sampling all species in a clade or geographic area. Here, I do the latter and provide the first complete description of the colour space occupied by an entire avifauna, the Australian landbirds. Australian landbirds are a good choice because they represent a moderately sized avifauna (~550 species, see Supplementary Table S1) with several radiations at a continental scale. I used reflectance spectrometry to measure reflectance of 17 standardized plumage patches (Supplementary Fig. S1) that cover all or most different colours on each specimen. Reflectance spectra were transformed into chromatic coordinates using visual models and plotted in avian visual space.

My general aim is to provide a quantitative description of the colour gamut of the entire Australian avifauna. Specifically, my analysis will include: (1) Determining how the colour space is filled, that is identifying which colours are common and which ones are rare, and how they are distributed through the colour space. (2) Quantifying the contribution of the major colour-production mechanisms to the filling of colour space. Bird colours are produced by a variety of mechanisms including pigment deposition (melanins, carotenoids, psittacofulvins), feather microstructure (structural colours) or a combination thereof. Previous work indicates that structural colours occupy a much larger portion of the colour space than pigmentary colours^8^, but the generality of these results is unclear. (3) Determining similarities and differences in the colour space of males and females: Sexual differences in ornamentation have been widely studied in birds (e.g. sexual dichromatism^10^,^11^) and variation in the strength of sexual selection seems to be the main correlate of sexual differences in coloration. While in general males are the more ornamented sex it is unclear whether this leads to unique male colours or whether the colour space is largely shared between the sexes. Finally, (4) I will estimate the contribution of the different taxonomic groups (e.g. parrots, passerines, etc.) to filling the chromatic space. In sum this study will assess how chromatic variation is partitioned at different levels, from the mechanisms of colour production, through sexes and to the major clades of Australian birds.

## Results

I obtained 46,559 reflectance spectra from 17 standardized plumage patches (Supplementary Fig. S1) of 2734 specimens belonging to 555 species, which represents 99% of the total number of Australian landbirds (559, following^12^, see Supplementary Table S1). For some species both sexes were missing from the collections used (n = 4), for others males (n = 6) or females (n = 10) were not available. Thus, complete coverage for both sexes was achieved for 539 species (96%). When analysing sex-specific colour variation the analysis was restricted to the subset of species with male and female colour information. I used visual models^7^ to compute chromatic coordinates, which define the position of each colour in colour space of birds. Visual sensitivities of birds can be classified into two main types: V-type and U-type, with the latter being more sensitive to UV wavelengths^13^. Among the birds included in my sample both visual systems are represented^14^. Whenever I had multiple measurements from different specimens of the same sex and species, I averaged chromatic coordinates prior to analysis.

**Figure 1:**
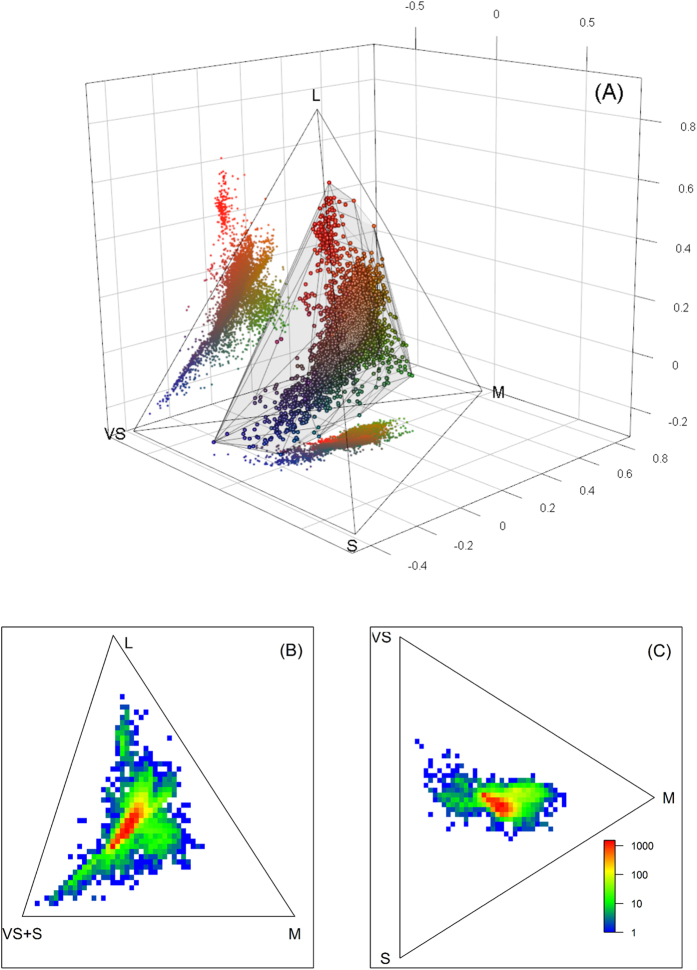
Plumage colours of Australian landbirds represented in U-type visual space. In (**A**) all colours are represented within the tetrahedral colour space of birds where symbols of different colours approximate human-perceived hues. The semi-transparent ‘shroud’ represents the convex-hull volume occupied by the entire sample. Rasterized versions in two dimensions, which correspond to the side and bottom ‘shadows’ in (**A**), are presented in (**B**)(side shadow) and (**C**) (bottom shadow) respectively. These two-dimensional projections of the three-dimensional variation in colour space would correspond to views from one side (**B**) or directly from above (**C**) the tetrahedron. Note that the lower left vertex of the triangle in (**B**) is labelled VS + S because, from this side view, it is not possible to separate colours based on their relative VS or S stimulation. Similarly, the L cone label has been omitted from (**C**) because relative stimulation of this cone type cannot be assessed in this projection. Warmer colours represent heavily occupied locations in colour space and lines represent the edges of the tetrahedron in two dimensions.

The plumage colours of Australian landbirds occupy 19% of the total theoretical avian colour space, based on a convex hull volume using U-type visual sensitivities (Fig. 1). If we repeat this analysis using V-type visual sensitivities this figure becomes smaller, 16%. While V-type visual sensitivities define a more constrained, smaller, chromatic volume all other patterns of colour variation are very similar for both types of visual systems. Hence, for simplicity I present the results for U-type visual sensitivities in the main text and the results for V-type visual sensitivities in the Supplementary Information.

Convex hull volumes are mostly composed of empty space (Fig. 1A) and hence tend to overestimate the occupied chromatic space, being particularly sensitive to the presence of outliers. To overcome this problem and for a more detailed assessment of colour distribution, I subdivided the chromatic space using a three-dimensional grid (Fig. 1 and Supplementary Fig. S2, see Methods for more detail). Within this grid each equilateral 3D cell represents a set of similar colours, providing a way of partitioning the continuous variation in colour space into discrete units (hereafter chromatic loci). The colour gamut of a particular subset can then be assessed by simply counting the number of chromatic loci occupied.

Examining plumage colour variation in visual space (Fig. 1) reveals an elongated-shaped cloud of points, with distinctive outcrops in specific directions which are occupied by the more extreme and saturated (or intense) colours of various hues. In human terms, these roughly contain saturated blue-violet, green, yellow and red colours (Fig. 1). For each chromatic locus I computed the number of plumage patches found within, which constitutes an index of how abundant colours in each category are. Colour abundance was not uniformly distributed throughout the avian chromatic gamut, and most plumage colours fell in a very restricted part of the chromatic volume. More than half of all measured colour patches were found in < 2% of chromatic loci which are located near the centre of the chromatic space (Fig. 1). These include colours ranging from white and black to grey and different shades of brown. Other colours are far less common in the sample and abundance rapidly decreases away from the dense core (Fig. 1).

### Mechanisms of colour production

**Figure 2:**
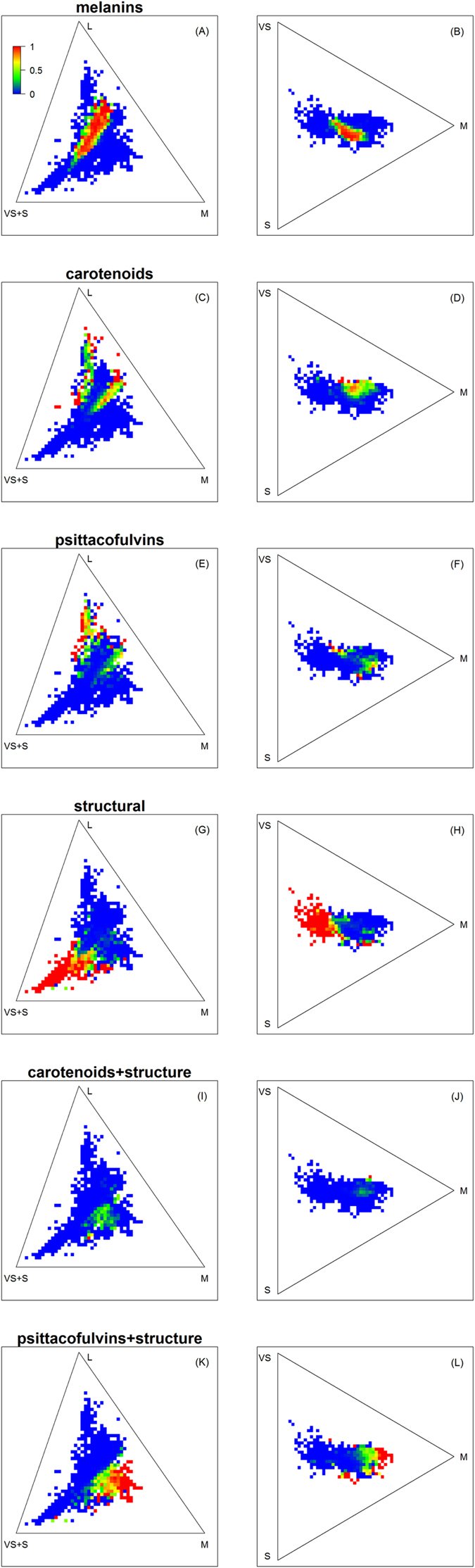
Plumage colours of Australian landbirds by type of colour production mechanism plotted in two dimensional rasterised colour space (see Fig. 1 and text for explanation). Melanins (**A,B**), carotenoids (**C,D**), psittacofulvins (**E,F**), structural colours (**G,H**), carotenoid + structure (**I,J**) and psittacofulvins + structure (**K,L**). Warmer colours represent locations in colour space with higher relative abundance for each colour-production mechanism and lines represent the edges of the tetrahedron in two dimensions.

Most (74.2%) of colours in the sample were classified as being melanin-based, 11.6% as carotenoid-based, 7.3% as structural, 2.6% as psittacofulvin-based, 1.1% as a combination of structure and carotenoids and 3.2% as a combination of structure and psittacofulvins. Despite the abundance of melanin-based colours they only occupied a small central portion of the avian colour gamut (6.8% of volume, 19.4% chromatic loci, Fig. 2A,B). The largest portion was occupied by structural colours (44.8% volume, 35.1% chromatic loci, Fig. 2G,H) followed by the combination of psittacofulvins and structure (26.5% volume, 27.7% chromatic loci, Fig. 2K,L), carotenoids (21.1% volume, 25.6% chromatic loci; Fig. 2C,D), psittacofulvins (17.7% volume, 22% chromatic loci; Fig. 2E,F), and the combination of carotenoids and structure (5.2% volume, 10% chromatic loci, Fig. 2I,J).

Structural colours also occupied the largest number of unique chromatic loci (23.6% of all occupied chromatic loci were only occupied by structural colours) followed by the combination of psittacofulvins and structure (18.5%), carotenoids (11.3%), psittacofulvins (9.8%), melanins (7.7%) and the combination of carotenoids and structure (2.7%). Melanins were most abundant in the centre of the chromatic space and extending towards longer wavelengths (rusty-red colours, Fig. 2A,B). Structural colours were overrepresented towards the shortwave end of the chromatic space (blue, violet, UV, Fig. 2G,H), psittacofulvins and carotenoids towards the long-wave end (red, yellow, Fig. 2C–F) while the combinations of psittacofulvins + structure and carotenoids + structure where mostly found towards middle wavelengths (greenish colours, Fig. 2I–L).

### Sex differences

**Figure 3:**
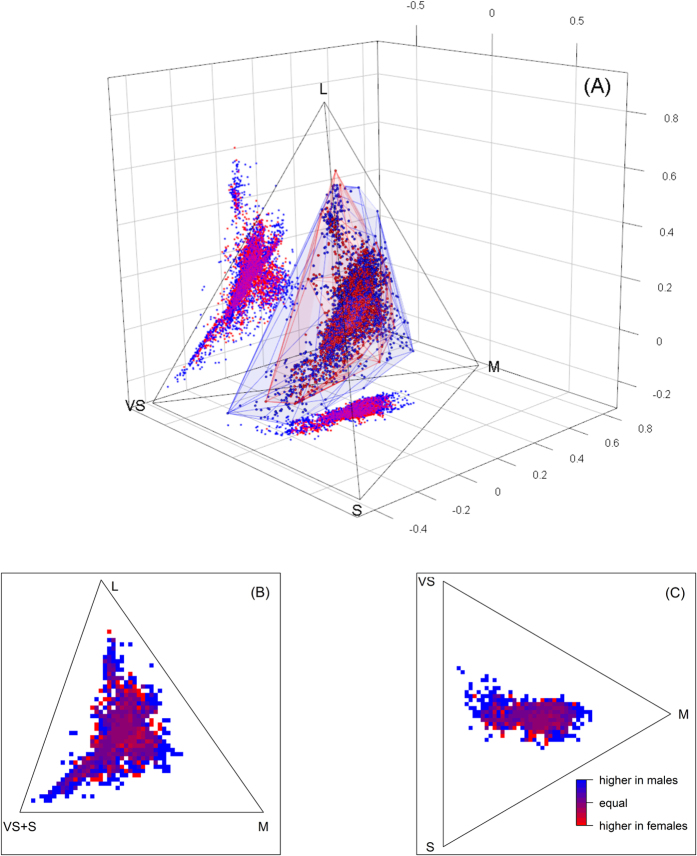
Male and female plumage colours of Australian landbirds. In (**A**) all males (blue and female (red) colours are represented within the tetrahedral colour space of birds. The semi-transparent ‘shrouds’ represents the convex-hull volume occupied by males and females. Rasterized versions in two dimensions, which correspond to the side and bottom ‘shadows’ in (**A**), are presented in (**B**) (side shadow) and (**C**) (bottom shadow) respectively. Colour shading represents whether colours are more abundant in male or female colour samples and lines represent the edges of the tetrahedron in two dimensions.

The chromatic volume occupied by males (0.062) is almost twice as large as the female volume (0.034) and surrounds it nearly completely (Fig. 3). The overlap in convex hull volume between the sexes comprises 99% of female- and 55% of male-occupied volume. In terms of chromatic loci females occupy 60% and males 76% of all chromatic loci; 47% of occupied loci are shared by the sexes, 15% are restricted to females and 37% to males. Bootstrapping (n = 10000) confirms that males occupy larger portions of the chromatic space than females (ratio female/male volume, median = 0.56, 2.5% quantile = 0.46, 97.5% quantile = 0.67; ratio female/male occupied chromatic loci: median = 0.76, 2.5% quantile = 0.71, 97.5% quantile = 0.81). Chromatic loci that are overrepresented among males are located in the periphery of the occupied chromatic space (Fig. 3).

### Taxonomic differences

**Figure 4:**
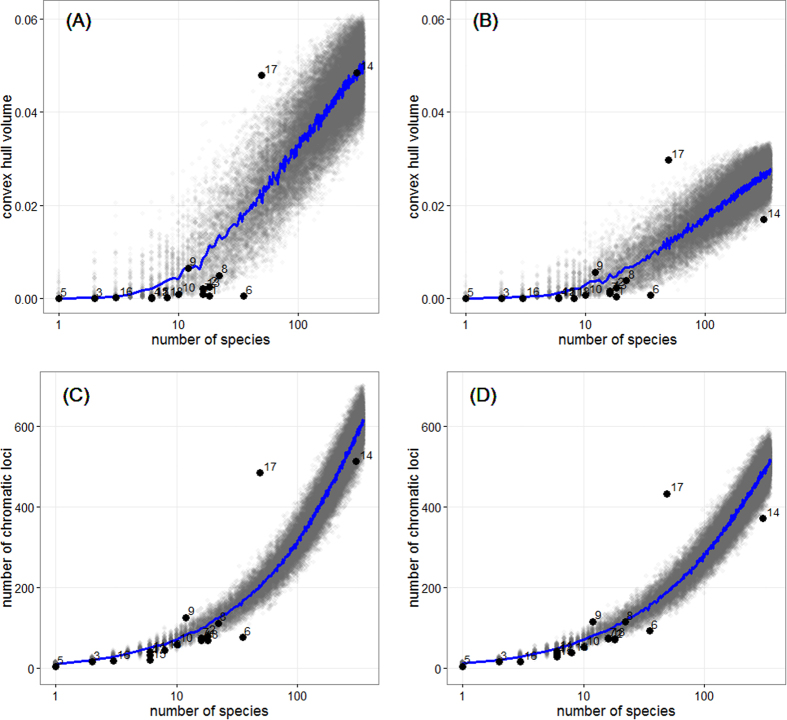
Taxonomic differences in colour volume (**A,B**) and number of occupied chromatic loci (**C,D**) for male (**A,C**) and female (**B,D**) colours. Depicted in blue are median expected values of colour diversity obtained by bootstrapping different number of species (for graphical purposes 100 samples for each species number), grey symbols indicate all bootstrapped values. Different avian orders are depicted using black symbols and numbers indicate order (see Tables 1 and 2 for reference key). Note that the x-axis is on a log_10_ scale to highlight differences between orders with few species.

Different orders occupy very different amounts of colour space. Male colours of Passeriformes (perching birds) and Psittaciformes (parrots and cockatoos) each occupy >70% of the total chromatic volume or >50% of the total number of occupied chromatic loci (Table 1, Fig. 4). These are however the most speciose orders in the sample and the occupied colour space is highly dependent on number of species (Fig. 4). If we compare each order with the expected colour volume based on bootstrapping the same number of species randomly selected from the entire avifauna (n = 10000 replicates in each case), Psittaciformes stand out as having much higher colour volume and number of occupied chromatic loci than expected (both males and females; Tables 1 and 2, Fig. 4). This is not the case for Passeriformes which fall within the expected range (chromatic volume males, Table 1) or significantly below (chromatic volume, females Table 2; number of chromatic loci both sexes, Tables 1 and 2). Among the other orders only Coraciiformes (kingfishers, Rainbow bee-eater, Dollarbird) have higher number of chromatic loci (but not colour volumes) than expected for their size (Tables 1 and 2). Most orders tend to have lower colour volumes and lower number of chromatic loci than expected (Tables 1 and 2), in particular Charadriiformes (shorebirds, gulls and terns, Fig. 4).

## Discussion

When we think about Australian birds, colourful species such as parrots, bowerbirds, fairy-wrens or finches come to mind. However, the colours of these gaudy species are a small minority and most Australian birds are not so well endowed. Colour variation is thus extremely uneven. Most colours are unremarkable and special colours, those that push the boundaries of colour space, are rare. Such inequality is also evident when comparing males and females or different clades: male colours almost completely surround female colours and a few selected clades of birds are much more colourful than expected for their number of species. Below I discuss these patterns starting with mechanisms of colour production.

By far the most common colours in Australian birds are those based on the deposition of melanins (nearly 75% of all measured plumage patches). Melanins are often considered the ancestral colour^8^. They mainly occupy the centre of the chromatic space, extending towards the long-wave rich sector of the chromatic space (e.g. rusty-red, Fig. 2). Melanins are presumably cheap to produce^15^ (but see^16^), can be synthesised by the birds themselves and may strengthen feathers^17^. Depositing them in the plumage may also be the best way to match the colours of a large part of natural backgrounds (such as soil, bark, etc.) for camouflage. These properties and functions may explain their abundance. However, most melanin-based colours are not very conspicuous and thus probably constitute poor visual signals. Visual signals are usually associated with other mechanisms of colour production. Deposition of carotenoids or psittacofulvins (in the case of parrots) produces colours rich in long wavelength reflectance (red, yellow). Structural colours are single-handedly responsible for all colours rich in short-wavelength reflectance (blue, violet, UV) and account for nearly half of the occupied colour space. Despite the fact that structural colours are in theory capable of producing almost any hue from red to UV^8^, most structural colours in Australian birds are concentrated at the shortwave end of the colour space (Fig. 2). Finally, the combination of structures with carotenoids or psittacofulvins creates colours rich in middle wavelengths (greens).

With the exception of carotenoids and psittacofulvins the different colour production mechanisms show relatively little overlap, filling colour space in complementary ways (Fig. 2). Psittacofulvins and carotenoids largely occupy similar portions of the colour space, but are never found together (psittacofulvins are restricted to parrots which do not deposit carotenoids in the plumage^18^). This broad scale complementarity means that - despite the clear differences in the amount of chromatic space occupied by the different mechanisms (Table 1, Fig. 2)^8^—high levels of colour space occupancy can only be achieved by producing colours using a variety of production mechanisms. Hence, differences in the occurrence of the various mechanisms of colour production may be partially responsible for the variation in occupied colour space by different clades or sexes.

Between half and a quarter of the colour space of Australian birds is occupied by male-only colours. Such a considerable difference between male and female colour space may be testament to the importance of sexual selection as a source for colour diversity. The intensity of sexual selection on males is a well-known correlate of sexual dichromatism in birds^11^,^19^ but to what extent differences in coloration between the sexes result in sex-specific colours is unclear. Males are usually more colourful than females^11^,^19^ but in many species males and females have similarly colourful plumages (mutually ornamented species^20^) suggesting that the range of male and female colours could be similar. My results indicate that, at least for the Australian avifauna, male colours of sexually dichromatic species tend to be more exaggerated (i.e. be found at the periphery of the colour space, Fig. 3) than female colours of mutually ornamented species or species with reversed sexual dichromatism (e.g. Eclectus parrots, *Eclectus roratus*^21^). Alternatively, species traditionally considered to be mutually ornamented may be more dimorphic than expected when differences in visual perception are taken into account^22^.

Is sexual selection responsible for differences in male and female colour space? While some cases of sexual dichromatism may be due to differences in habitat use, which require of different cryptic colours^23^, such differences are relatively subtle and uncommon. Most likely extreme, male-exclusive, colours are a consequence of their heightened visual signalling needs due to strong sexual selection^24^. Male-only colours are most common towards the edges of colour space (Fig. 3). This makes them rare colours and colours that most likely will contrast strongly against common natural backgrounds (which are found towards the centre of colour space^25^). These extreme colours may represent a particularly costly expression of visual signals, either because the production costs entailed (for example deposition of high quantities of limiting carotenoids^26^) or other ongoing costs such as maintenance^27^,^28^ or increased predation risk^29^. Alternatively, or in addition, extreme colours could constitute the endpoints of arbitrary processes such as Fisher-like processes of sexual selection^30^. Regardless of the mechanism, sexual selection is largely responsible for at least a third of the occupied colour space among Australian birds. This figure is certainly an underestimate given that sexual selection also acts on females^31^.

Sexual differences in colour space occupancy are particularly obvious in clades that occupy large amounts of colour space, namely parrots (Psittaciformes) and passerines (Passeriformes) (Fig. 4). These are also the most speciose orders of Australian landbirds (Table 1). But while bootstrap revealed that both male and female parrots occupy larger than expected colour space for their number of species, Passeriformes fall either within the expected range (males) or well below it (females, Fig. 4). Most other orders have expected or lower than expected levels of colour diversity for their number of species (convex hull volume and number of chromatic loci, Fig. 4). Lower than expected values were particularly evident for Charadriiformes (plovers, sandpipers, gulls, terns, Fig. 4). Besides parrots only one other order of Australian birds (Coraciiformes, which includes kingfishers, dollarbird and rainbow bee-eater) showed higher than expected colour diversity but this effect was much smaller (Fig. 4). While parrots have long been regarded as a particularly colourful group of birds^32^ the present study constitutes the first quantitative test that confirms this observation.

Why are Australian parrots so colourful? Parrots are special because they use a unique type of pigments, psittacofulvins, to colour their feathers^32^. In the absence of carotenoids, psittacofulvins may be the parrot’s alternative to produce longwave-rich colours such as red, yellow or -in combination with structural colours- green hues^8^. However, being synthesised endogenously, parrots may be able to deposit higher concentrations and produce more intense colours than comparable carotenoid-based colours which require pigments to be obtained from the diet. However, the overall overlap in colours produced by carotenoids and psittacofulvins^8^ (and this study, Fig. 2) argues against the idea that psittacofulvins alone could explain the high levels of colour diversity in this clade. Varied coloration could also be due to strong selection favouring visual signals. Sexual selection on males is not thought to be particularly strong in parrots and many species are mutually ornamented^32^. However, my data indicate that sex differences in colour diversity are still noticeably large in Australian parrots (Fig. 4), so male-biased sexual selection may be more prominent than thought in this clade. Alternatively, group-living and long-term social interactions may select for diverse colours to signal status, age or identity^33^,^34^. Finally, given that most species are hole-nesters, safe nesting sites may have relaxed natural selection which limit the evolution of conspicuous colours in other clades of birds^35^,^36^. The strong differences in colour space occupation between clades of birds contrast with the lack of such differences for fruit colours, which overlap broadly between clades and show little phylogenetic signal^9^.

In conclusion, a nearly complete assessment of the colour gamut of an avifauna reveals striking inequalities in the distribution of colour diversity. Most of the colour diversity is due to comparatively few species that tend to be found clumped in certain branches of the phylogenetic tree. But how representative are Australian bird colours of the rest of birds? One of the main results of the previous assessment of the avian colour gamut by Stoddard and Prum^8^ was that bird colours occupied only a limited portion (30%) of the colour space. Australian bird colours occupy an even smaller section (19%); does this mean that they are particularly dull? Rather, the most likely reason for this discrepancy is that different criteria were used to select the samples. While in the current study species were not selected (beyond the fact that they had to be found in the study area and be available at the collections), Stoddard and Prum^8^ selected species to cover the most extreme plumage colours and all mechanisms of colour production. Thus their sample probably constitutes a good approximation of the total range of colours of birds (which was their intention), and most regional avifaunas will necessarily be a subset of this range. Some mechanisms of colour production will be completely missing (e.g. turacins and turacoverdins in Australia), while others may be under- or over-represented (in particular melanins). Despite these differences, I suspect that the general patterns uncovered here, such as the preponderance of rather dull melanin-based colours will apply to most, if not all avifaunas. For example Bailey^37^ assessed colour variation (based on colour plates) of the North- and Central-American avifauna and concluded that “…’dull’ species predominate everywhere”, a statement that could apply equally well to Australian birds. Less clear is whether the colour gamut of other avifaunas will be strongly dominated by specific clades of birds. Do other clades of birds reach similar levels of colourfulness as parrots? Only with comparable data from other continents will it be possible to put these results into context and answer the final question: how colourful are Australian birds?

## Methods

### Study species

Australian landbirds were defined as those bird species that regularly breed in the Australian mainland or Tasmania excluding species endemic to other small islands (e.g. Lord Howe Island, Christmas Island, etc.). Pelagic species such as petrels, albatrosses, gannets, etc. were excluded but coastal species and other aquatic species (e.g. terns, gulls, ducks, cormorants, etc.) were included. Non-breeding migratory species were also excluded. Based on the nomenclature of Christidis and Boles^12^ this yielded a list of 559 species to be measured (see Supplementary Table S1). Not all these species were represented at the collections which resulted in some not being measured (see Results and Supplementary Table S1).

Reflectance spectra were obtained from museum specimens housed at the ornithological collections of the Melbourne Museum and the Australian National Wildlife Collection in Canberra. For each species I aimed to sample 3 males and 3 females but this was not always possible (number of sampled specimens: males, mean = 2.62, range = 1–6; females, mean = 2.41, range = 1–6). When possible I sampled specimens from the same subspecies or general geographic area. Only specimens with well-preserved plumage were measured. For those species that moult into a different breeding plumage, only specimens in breeding plumage were measured. Reflectance spectra were collected from 17 homologous plumage patches distributed over the entire body (Supplementary Fig. S1). Only plumage colours were measured. The bare patches of skin present in some species were not measured as the colour of fleshy parts fades rapidly after death. Reflectance spectra between 300 and 700 nm were obtained used an Avaspec 2048 spectrometer (Avantes, Eerbeek, Netherlands) connected through a bifurcated fibre optics cable to a pulsed Xenon light source (Avalight XE). The measuring probe was fitted at the end with a black plastic cylinder to standardize measuring distance and exclude ambient light. Measuring and illumination angles were both 90°. Reflectance was computed relative to a WS-2 white standard.

### Visual Modelling

Reflectance spectra were down-sampled to 5 nm steps and imported into R computing environment^38^. We used the approach of Stoddard and Prum^8^ to obtain chromatic coordinates (xyz) which define the position of each spectrum in the visual space of birds. Cone quantum catches were obtained using custom-made scripts^39^ based on^40^ and converted to chromatic coordinates using formulas outlined in Kelber *et al.*^41^. The chromatic space is usually represented as a tetrahedron, where each vertex corresponds to the sole stimulation of a single cone. In this study the tetrahedron is oriented such as the L cone is found at the apex (Fig. 1). Given that my aim was to assess colour variation I used an ideal flat irradiance spectrum^8^ (although results are very similar if other irradiances are used).

In birds the visual sensitivity functions of the four single cones used in colour vision fall into two major groups (U- and V-type) that differ mainly in their sensitivity to shorter wavelengths^13^ (blue to UV). While differences in visual sensitivities do lead to differences in colour perception^42^ here I focus on analysing colour variation irrespective of visual system variation. I modelled plumage colours from all species using both U- and V-type eyes (results for V-type eyes are provided in the Supplementary Information). I used visual sensitivity functions from^43^ which have been obtained by computing the average for those species with suitable information in each group.

### Assigning colours to colour-producing mechanisms

Reflectance spectra were assigned to six broad colour production mechanisms (melanins, carotenoids, psittacofulvins, structural, carotenoids + structural, psittacofulvins + structural) based on specific aspects of spectral shape^44^,^45^. Spectra were considered melanin-based if they presented (a) uniform flat reflectance curves throughout most of the wavelength range (these could include reduced reflectance towards the ultraviolet), or (b) monotonically increases in reflectance towards longer wavelengths without a clear maximum^45^ (Supplementary Fig. S3). Note that ‘white’ spectra which are largely unpigmented feathers were also included in this category. This was done for the sake of convenience given that determining what is white and what not (e.g. light grey) is essentially arbitrary. Including white spectra with melanin-based colours is unlikely to have biased results given the small amount of colour space occupied by white plumage^8^.

Spectra were considered carotenoid-based if they presented (a) a trough in reflectance at around 450 nm with characteristic absorption peaks around that value^44^(Supplementary Fig. S4) and higher reflectance at longer wavelengths, or (b) low reflectance at shorter wavelengths which increased towards 700 nm but showing a characteristic ‘shoulder’ at longer wavelengths, resulting in a sigmoid-shaped spectrum^45^ (Supplementary Fig S4). Purple reflectance spectra (Supplementary Fig. S4) detected in some *Ptilinopus* doves are also carotenoid-based^46^. If these spectral characteristics were observed in species of the order Psittaciformes, which replace carotenoid pigments with psittacofulvins, those plumage patches were assigned to the category psittacofulvin-based^45^ (Supplementary Fig. S5). Structural colours were identified by reflectance spectra showing bell-shaped curves with discrete maxima throughout the wavelength range (Supplementary Fig. S6). In some cases (iridescent colours) reflectance spectra presented multiple peaks (Supplementary Fig. S6). Spectra that combined a blue structure with deposition of yellow carotenoid^44^ or psittacofulvin pigments were allocated to the carotenoids + structure or psittacofulvins + structure categories (Supplementary Figs. S7-8). These spectra showed a trough around 450 nm (with characteristic absorption peaks, see above), a peak between 500 and 600 nm, followed by another trough at longer wavelengths.

I assessed the accuracy of my assignment of colour-production mechanisms by comparing my assessment against recent summaries for the different mechanisms (carotenoids^47^, structural colours^48^, melanins^45^, psittacofulvins^18^,^32^) and in case of doubt against the primary literature cited therein. In all cases (n = 47 species) my assessment matched that of previous studies. For most species included in this study there are no detailed studies on the nature of the colour-production mechanism. However, in those cases where closely related species with similar colours had been studied (n = 17 species), the assigned colour-production mechanism matched my assignment as well.

Despite the agreement, this way to categorize colours into different mechanisms is certainly a rough estimate at best. For example given that almost all feathers will contain some amount of melanins and that melanins are essential for the production of other colours (e.g. structural colours^49^) the classification scheme used will have underestimated the true effects of melanins. In addition most pigmentary colours rely on the reflectance properties of keratin which provide a white base colour^50^. Finally, while most colours can be confidently classified into one category, there are intermediate cases where assessing the main mechanisms contributing to colour production is difficult. This difficulty is particularly evident when dealing with colours that are produced by a combination of structure and carotenoids or psittacofulvins. As a result, this approach only broadly separates the main types of colours. More detailed analyses will require in-depth quantification of the contribution of each mechanism to colour production in a case-by-case basis. Meanwhile, the categories proposed here constitute a first step towards quantifying the contribution of each colour-producing mechanism to the avian colour gamut.

### Data analysis

The total range of colours can be estimated computing the volume of a convex hull that encloses all points in a sample^8^. Convex hull volume and convex hull volume overlap between pairs of volumes were computed using the R packages ‘pavo’^51^ and ‘geometry’^52^. However, convex hull volumes tend to overestimate occupied space because they include large sections of empty space and are thus particularly sensitive to the presence of extreme samples. To overcome this problem and for a more detailed assessment of colour distribution, I subdivided the chromatic space using a three-dimensional grid with the R package ‘raster’^53^. This was done in a series of steps. (1) The package ‘raster’ is used to create two 2-dimensional grid systems, using the yz and yx axes respectively (the choice of planes is arbitrary). (2) After rasterization each colour can be assigned to a specific 2D cell on each of the planes, and the identity and coordinates of each cell are associated with each colour. (3) Intersecting the two 2D grid systems defines cells in a 3D grid (Fig. S2), and combining the cell identities of both grid systems identifies the chromatic loci in the 3D grid. Each 3D cell (dimensions: 0.022 × 0.022 × 0.022) then represents a chromatic locus, providing a way of partitioning the continuous variation in colour space into discrete units (see Supplementary Fig. S2). The colour gamut of a particular subset can then be assessed by simply counting the number of colour loci occupied. Convex hull volume and number of occupied chromatic loci are positively correlated (for example for the bootstrapped male data used in Fig. 4, Pearson’s r = 0.9, p < 0.001) but convex hull volume shows more noise due to the strong influence of outliers (compare Fig. 4A with 4 C for example). However, partitioning the colour space into chromatic loci has a further advantage, since for each cell I also compute the number of plumage patches found within, which constitutes an index of how common colours in each category are.

I used bootstrap to assess observed and expected differences in occupied colour volume or number of chromatic loci. To determine the difference between males and females 10000 random sets of species were sampled with replacement from the entire sample for which male and female colours measurement were available (n = 539 species). For each random set of species I obtained colour volume and number of chromatic loci for males and females and computed the ratio between females and males (female/male). The bootstrapped distribution of female/male ratios was then compared to the null hypothesis of equal levels of colour diversity (ratio = 1). To assess whether bird orders had higher or lower colour diversity than expected for their number of species I compared the observed values for each order with 10000 bootstrapped samples with the same number of species. This was done separately for males and females.

## Additional Information

**How to cite this article**: Delhey, K. The colour of an avifauna: A quantitative analysis of the colour of Australian birds. *Sci. Rep.* **5**, 18514; doi: 10.1038/srep18514 (2015).

## Supplementary Material

Supplementary Information

## Figures and Tables

**Table 1:** Comparing observed values of male colour diversity for each bird order (% occupied convex hull volume and chromatic loci) with the expected values obtained from bootstrapping the same number of random species from the entire sample of male colours.

	ORDER	n	% of male convex hull volume				% of male occupied chromatic loci			
			observed	2.5% quantile bootstrap	97.5% quantile bootstrap	p-value	observed	2.5% quantile bootstrap	97.5% quantile bootstrap	p-value
1	ACCIPITRIFORMES	18	0.83	2.77	40.31	0.0002	7.34	8.18	14.99	0.0064
2	ANSERIFORMES	18	3.87	2.77	40.31	0.0982	8.39	8.18	14.99	0.0684
3	APODIFORMES	2	0.10	0.02	10.97	0.3818	1.68	1.26	3.04	0.306
4	CAPRIMULGIFORMES	6	0.12	0.33	22.23	0.0020	3.35	3.67	7.23	0.0164
5	CASUARIIFORMES	1	0.00	0.00	5.69	0.0090	0.42	0.52	1.68	0.041
6	CHARADRIIFORMES	35	0.86	10.11	53.00	0.0000	8.07	13.10	22.33	<0.0001
7	CICONIIFORMES	35	1.53	2.30	38.33	0.0124	7.86	7.65	14.05	0.0878
8	COLUMBIFORMES	22	7.96	4.39	44.01	0.2052	11.74	9.54	16.98	0.5612
9	CORACIIFORMES	12	10.52	1.37	33.93	0.9756	13.21	6.18	11.64	0.0036
10	CUCULIFORMES	10	1.57	0.99	30.25	0.1476	6.29	5.45	10.38	0.277
11	FALCONIFORMES	6	0.16	0.33	22.23	0.0058	4.30	3.67	7.23	0.2768
12	GALLIFORMES	6	0.32	0.33	22.23	0.0474	3.35	3.67	7.23	0.0164
13	GRUIFORMES	16	3.44	2.30	38.33	0.1176	7.23	7.65	14.05	0.0246
14	PASSERIFORMES	305	77.71	61.06	92.31	0.9726	53.77	52.94	67.82	0.0878
15	PHALACROCORACIFORMES	6	0.21	0.33	22.23	0.0146	2.20	3.67	7.23	0.0002
16	PODICIPEDIFORMES	3	0.21	0.07	13.91	0.3184	1.99	1.99	4.30	0.0606
17	PSITTACIFORMES	49	76.69	16.30	59.32	0.0006	50.73	16.35	26.94	<0.0001
18	STRIGIFORMES	8	0.27	0.65	26.63	0.0050	4.72	4.61	8.91	0.0752

P-values are two-tailed and represent the proportion of bootstrapped values more extreme than the observed value.

**Table 2:** Comparing observed values of female colour diversity for each bird order (% occupied convex hull volume and chromatic loci) with the expected values obtained from bootstrapping the same number of random species from the entire sample of female colours.

	Order	n	% of female convex hull volume				% of female occupied chromatic loci			
			observed	2.5% quantile bootstrap	97.5% quantile bootstrap	p-value	observed	2.5% quantile bootstrap	97.5% quantile bootstrap	p-value
1	ACCIPITRIFORMES	18	1.07	3.35	39.23	<0.0001	9.53	9.92	18.02	0.0286
2	ANSERIFORMES	18	6.75	3.35	39.23	0.2898	9.27	9.92	18.02	0.0170
3	APODIFORMES	2	0.12	0.05	10.77	0.2810	2.09	1.70	3.79	0.2468
4	CAPRIMULGIFORMES	6	0.38	0.51	21.74	0.0176	4.57	4.70	8.88	0.0432
5	CASUARIIFORMES	1	0.00	0.00	3.80	0.0044	0.65	0.78	2.09	0.0380
6	CHARADRIIFORMES	35	2.03	9.30	51.63	<0.0001	12.14	15.40	25.85	0.0004
7	CICONIIFORMES	35	3.39	2.93	36.96	0.0828	9.79	9.27	16.84	0.1152
8	COLUMBIFORMES	22	11.37	4.52	42.88	0.4458	15.01	11.36	20.10	0.9150
9	CORACIIFORMES	12	16.54	1.88	33.18	0.5142	15.14	7.57	14.10	0.0106
10	CUCULIFORMES	10	2.05	1.32	28.85	0.1418	6.92	6.79	12.53	0.0804
11	FALCONIFORMES	6	0.52	0.51	21.74	0.0506	5.48	4.70	8.88	0.3410
12	GALLIFORMES	6	0.24	0.51	21.74	0.0028	4.70	4.70	8.88	0.0650
13	GRUIFORMES	16	4.50	2.93	36.96	0.1818	9.66	9.27	16.84	0.0992
14	PASSERIFORMES	305	49.59	59.66	91.91	0.0010	48.43	55.48	70.89	0.0002
15	PHALACROCORACIFORMES	6	0.24	0.51	21.74	0.0026	3.66	4.70	8.88	0.0012
16	PODICIPEDIFORMES	3	0.22	0.12	14.49	0.1732	2.22	2.61	5.22	0.0134
17	PSITTACIFORMES	49	86.81	14.94	58.19	<0.0001	56.53	18.93	30.94	<0.0001
18	STRIGIFORMES	8	0.20	0.84	26.38	<0.0001	4.96	5.74	10.97	0.0060

P-values are two-tailed and represent the proportion of bootstrapped values more extreme than the observed value.
