# Supplementary material for: The colour of an avifauna: A quantitative analysis of the colour of Australian birds
Source: Sci Rep. 2015 Dec 18;5:18514. doi: 10.1038/srep18514 (PMC4683462; doi:10.1038/srep18514)
Supplement: Supplementary Information [file srep18514-s1.pdf]

Supplementary Information

The colour of an avifauna: a quantitative analysis of the colour of Australian birds

Kaspar Delhey – Monash University, Australia

Table of Contents (click to follow link)

Plumage patches 2
Figure S1 2
List of species 3
Table S1. 3
3D grid in visual space 16
Figure S2 16
Examples of reflectance spectra 17
Figure S3 17
Figure S4 18
Figure S5 19
Figure S6 20
Figure S7 21
Figure S8 22
Summary of results for V-type visual systems 23
Table S2. 23
Table S3. 25
Table S4. 26

Plumage patches

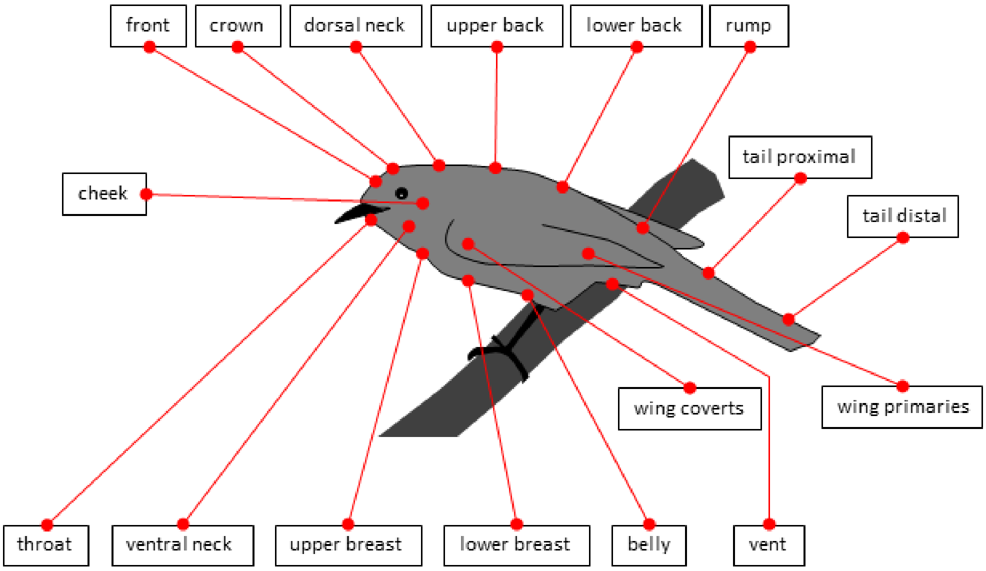

Figure S1. **Plumage patches measured in this study. Drawing by the author.**

List of species

Table S1. List of species included in this study, including number of female and male specimens measured per species. Taxonomy follows Christidis and Boles (2008).

Scientific name	English name	order	family	females	males
Acanthiza apicalis	Inland Thornbill	PASSERIFORMES	Acanthizidae	3	3
Acanthiza chrysorrhoa	Yellow-rumped Thornbill	PASSERIFORMES	Acanthizidae	3	3
Acanthiza ewingii	Tasmanian Thornbill	PASSERIFORMES	Acanthizidae	1	2
Acanthiza inornata	Western Thornbill	PASSERIFORMES	Acanthizidae	3	3
Acanthiza iredalei	Slender-billed Thornbill	PASSERIFORMES	Acanthizidae	3	1
Acanthiza katherina	Mountain Thornbill	PASSERIFORMES	Acanthizidae	2	2
Acanthiza lineata	Striated Thornbill	PASSERIFORMES	Acanthizidae	3	3
Acanthornis magna	Scrubtit	PASSERIFORMES	Acanthizidae	1	1
Acanthiza nana	Yellow Thornbill	PASSERIFORMES	Acanthizidae	2	3
Acanthiza pusilla	Brown Thornbill	PASSERIFORMES	Acanthizidae	3	3
Acanthiza reguloides	Buff-rumped Thornbill	PASSERIFORMES	Acanthizidae	3	3
Acanthiza robustirostris	Slaty-backed Thornbill	PASSERIFORMES	Acanthizidae	1	1
Acanthagenys rufogularis	Spiny-cheeked Honeyeater	PASSERIFORMES	Meliphagidae	3	3
Acanthorhynchus superciliosus	Western Spinebill	PASSERIFORMES	Meliphagidae	3	3
Acanthorhynchus tenuirostris	Eastern Spinebill	PASSERIFORMES	Meliphagidae	3	3
Acanthiza uropygialis	Chestnut-rumped Thornbill	PASSERIFORMES	Acanthizidae	3	3
Accipiter cirrocephalus	Collared Sparrowhawk	ACCIPITRIFORMES	Accipitridae	3	3
Accipiter fasciatus	Brown Goshawk	ACCIPITRIFORMES	Accipitridae	3	3
Accipiter novaehollandiae	Grey Goshawk	ACCIPITRIFORMES	Accipitridae	2	2
Acrocephalus australis	Australian Reed-Warbler	PASSERIFORMES	Acrocephalidae	1	3
Aegotheles cristatus	Australian Owlet-nightjar	APODIFORMES	Aegothelidae	3	3
Aerodramus terraereginae	Australian Swiftlet	APODIFORMES	Apodidae	3	3
Ailuroedus crassirostris	Green Catbird	PASSERIFORMES	Ptilonorhynchidae	2	3
Ailuroedus melanotis	Spotted Catbird	PASSERIFORMES	Ptilonorhynchidae	3	3
Alectura lathami	Australian Brush-turkey	GALLIFORMES	Megapodiidae	2	2
Alisterus scapularis	Australian King-Parrot	PSITTACIFORMES	Psittacidae	3	3
Amaurornis moluccana	Pale-vented Bush-hen	GRUIFORMES	Rallidae	1	2
Amblyornis newtonianus	Golden Bowerbird	PASSERIFORMES	Ptilonorhynchidae	3	3
Amytornis ballarae	Kalkadoon Grasswren	PASSERIFORMES	Maluridae	3	3
Amytornis barbatus	Grey Grasswren	PASSERIFORMES	Maluridae	2	3
Amytornis dorotheae	Carpentarian Grasswren	PASSERIFORMES	Maluridae	5	5
Amytornis goyderi	Eyrean Grasswren	PASSERIFORMES	Maluridae	3	6
Amytornis housei	Black Grasswren	PASSERIFORMES	Maluridae	1	1
Amytornis merrotsyi	Short-tailed Grasswren	PASSERIFORMES	Maluridae	3	2
Amytornis purnelli	Dusky Grasswren	PASSERIFORMES	Maluridae	3	3
Amytornis striatus	Striated Grasswren	PASSERIFORMES	Maluridae	2	3
Amytornis textilis	Thick-billed Grasswren	PASSERIFORMES	Maluridae	3	3
Amytornis woodwardi	White-throated Grasswren	PASSERIFORMES	Maluridae	2	3
Anas castanea	Chestnut Teal	ANSERIFORMES	Anatidae	3	3

Anas gracilis	Grey Teal	ANSERIFORMES	Anatidae	3	3
Anas rhynchotis	Australasian Shoveler	ANSERIFORMES	Anatidae	3	2
Anas superciliosa	Pacific Black Duck	ANSERIFORMES	Anatidae	3	3
Anhinga novaehollandiae	Australasian Darter	PHALACROCORACIFORMES	Anhingidae	3	3
Anseranas semipalmata	Magpie Goose	ANSERIFORMES	Anseranatidae	3	2
Anthochaera carunculata	Red Wattlebird	PASSERIFORMES	Meliphagidae	3	3
Anthochaera chrysoptera	Little Wattlebird	PASSERIFORMES	Meliphagidae	3	3
Anthochaera lunulata	Western Wattlebird	PASSERIFORMES	Meliphagidae	3	2
Anthus novaeseelandiae	Australasian Pipit	PASSERIFORMES	Motacillidae	3	3
Anthochaera paradoxa	Yellow Wattlebird	PASSERIFORMES	Meliphagidae	2	3
Anthochaera phrygia	Regent Honeyeater	PASSERIFORMES	Meliphagidae	3	3
Aphelocephala leucopsis	Southern Whiteface	PASSERIFORMES	Acanthizidae	3	3
Aphelocephala nigricincta	Banded Whiteface	PASSERIFORMES	Acanthizidae	1	2
Aphelocephala pectoralis	Chestnut-breasted Whiteface	PASSERIFORMES	Acanthizidae	1	1
Aplonis metallica	Metallic Starling	PASSERIFORMES	Sturnidae	3	3
Aprosmictus erythropterus	Red-winged Parrot	PSITTACIFORMES	Psittacidae	3	3
Aquila audax	Wedge-tailed Eagle	ACCIPITRIFORMES	Accipitridae	2	3
Ardeotis australis	Australian Bustard	GRUIFORMES	Otididae	2	1
Ardea ibis	Cattle Egret	CICONIIFORMES	Ardeidae	1	0
Ardea intermedia	Intermediate Egret	CICONIIFORMES	Ardeidae	1	3
Ardea pacifica	White-necked Heron	CICONIIFORMES	Ardeidae	3	1
Arses kaupi	Pied Monarch	PASSERIFORMES	Monarchidae	2	1
Arses telescopthalmus	Frilled Monarch	PASSERIFORMES	Monarchidae	3	1
Artamus cinereus	Black-faced Woodswallow	PASSERIFORMES	Artamidae	3	3
Artamus cyanopterus	Dusky Woodswallow	PASSERIFORMES	Artamidae	3	3
Artamus leucorynchus	White-breasted Woodswallow	PASSERIFORMES	Artamidae	3	1
Artamus minor	Little Woodswallow	PASSERIFORMES	Artamidae	3	3
Artamus personatus	Masked Woodswallow	PASSERIFORMES	Artamidae	3	3
Artamus superciliosus	White-browed Woodswallow	PASSERIFORMES	Artamidae	3	3
Ashbyia lovensis	Gibberbird	PASSERIFORMES	Meliphagidae	3	1
Atrichornis clamosus	Noisy Scrub-bird	PASSERIFORMES	Atrichornithidae	0	1
Atrichornis rufescens	Rufous Scrub-bird	PASSERIFORMES	Atrichornithidae	1	3
Aviceda subcristata	Pacific Baza	ACCIPITRIFORMES	Accipitridae	2	3
Aythya australis	Hardhead	ANSERIFORMES	Anatidae	3	3
Barnardius zonarius	Australian Ringneck	PSITTACIFORMES	Psittacidae	3	3
Biziura lobata	Musk Duck	ANSERIFORMES	Anatidae	2	3
Botaurus poiciloptilus	Australasian Bittern	CICONIIFORMES	Ardeidae	3	3
Burhinus grallarius	Bush Stone-curlew	CHARADRIIFORMES	Burhinidae	1	1
Butorides striata	Striated Heron	CICONIIFORMES	Ardeidae	2	3
Cacomantis castaneiventris	Chestnut-breasted Cuckoo	CUCULIFORMES	Cuculidae	0	3
Cacomantis flabelliformis	Fan-tailed Cuckoo	CUCULIFORMES	Cuculidae	3	3
Cacatua galerita	Sulphur-crested Cockatoo	PSITTACIFORMES	Cacatuidae	3	1
Cacomantis pallidus	Pallid Cuckoo	CUCULIFORMES	Cuculidae	3	3
Cacatua pastinator	Western Corella	PSITTACIFORMES	Cacatuidae	2	3
Cacatua sanguinea	Little Corella	PSITTACIFORMES	Cacatuidae	3	3
Cacatua tenuirostris	Long-billed Corella	PSITTACIFORMES	Cacatuidae	3	3

Cacomantis variolosus	Brush Cuckoo	CUCULIFORMES	Cuculidae	2	3
Calyptorhynchus banksii	Red-tailed Black-Cockatoo	PSITTACIFORMES	Cacatuidae	3	3
Calyptorhynchus baudinii	Baudin's Black-Cockatoo	PSITTACIFORMES	Cacatuidae	2	2
Calamanthus campestris	Rufous Fieldwren	PASSERIFORMES	Acanthizidae	1	1
Callocephalon fimbriatum	Gang-gang Cockatoo	PSITTACIFORMES	Cacatuidae	3	3
Calamanthus fuliginosus	Striated Fieldwren	PASSERIFORMES	Acanthizidae	3	3
Calyptorhynchus funereus	Yellow-tailed Black-Cockatoo	PSITTACIFORMES	Cacatuidae	3	2
Calyptorhynchus lathami	Glossy Black-Cockatoo	PSITTACIFORMES	Cacatuidae	3	3
Calyptorhynchus latirostris	Carnaby's Black-Cockatoo	PSITTACIFORMES	Cacatuidae	2	3
Caprimulgus macrurus	Large-tailed Nightjar	CAPRIMULGIFORMES	Caprimulgidae	1	2
Carterornis leucotis	White-eared Monarch	PASSERIFORMES	Monarchidae	1	3
Casuarius casuarius	Southern Cassowary	CASUARIIFORMES	Casuariidae	2	1
Centropus phasianinus	Pheasant Coucal	CUCULIFORMES	Cuculidae	2	3
Cereopsis novaehollandiae	Cape Barren Goose	ANSERIFORMES	Anatidae	1	2
Certhionyx variegatus	Pied Honeyeater	PASSERIFORMES	Meliphagidae	3	3
Ceyx azureus	Azure Kingfisher	CORACIIFORMES	Alcedinidae	3	3
Ceyx pusilla	Little Kingfisher	CORACIIFORMES	Alcedinidae	3	3
Charadrius australis	Inland Dotterel	CHARADRIIFORMES	Charadriidae	3	2
Chalcites basalis	Horsfield's Bronze-Cuckoo	CUCULIFORMES	Cuculidae	3	3
Chalcophaps indica	Emerald Dove	COLUMBIFORMES	Columbidae	3	3
Chalcites lucidus	Shining Bronze-Cuckoo	CUCULIFORMES	Cuculidae	3	3
Chalcites minutillus	Little Bronze-Cuckoo	CUCULIFORMES	Cuculidae	1	3
Chalcites osculans	Black-eared Cuckoo	CUCULIFORMES	Cuculidae	3	3
Charadrius ruficapillus	Red-capped Plover	CHARADRIIFORMES	Charadriidae	3	3
Chenonetta jubata	Australian Wood Duck	ANSERIFORMES	Anatidae	3	3
Cheramoeca leucosterna	White-backed Swallow	PASSERIFORMES	Hirundinidae	3	2
Chlidonias hybrida	Whiskered Tern	CHARADRIIFORMES	Laridae	3	3
Chroicocephalus novaehollandiae	Silver Gull	CHARADRIIFORMES	Laridae	3	3
Chthonicola sagittata	Speckled Warbler	PASSERIFORMES	Acanthizidae	6	6
Cinclosoma castaneothorax	Chestnut-breasted Quail-thrush	PASSERIFORMES	Psophodidae	1	1
Cinclosoma castanotum	Chestnut Quail-thrush	PASSERIFORMES	Psophodidae	1	2
Cinclosoma cinnamomeum	Cinnamon Quail-thrush	PASSERIFORMES	Psophodidae	1	2
Cincloramphus cruralis	Brown Songlark	PASSERIFORMES	Megaluridae	1	3
Cincloramphus mathewsi	Rufous Songlark	PASSERIFORMES	Megaluridae	1	3
Cinclosoma punctatum	Spotted Quail-thrush	PASSERIFORMES	Psophodidae	3	3
Circus approximans	Swamp Harrier	ACCIPITRIFORMES	Accipitridae	1	1
Circus assimilis	Spotted Harrier	ACCIPITRIFORMES	Accipitridae	2	3
Cisticola exilis	Golden-headed Cisticola	PASSERIFORMES	Cisticolidae	2	2
Cisticola juncidis	Zitting Cisticola	PASSERIFORMES	Cisticolidae	3	2
Cissomela pectoralis	Banded Honeyeater	PASSERIFORMES	Meliphagidae	2	3
Cladorhynchus leucocephalus	Banded Stilt	CHARADRIIFORMES	Recurvirostridae	3	3
Climacteris affinis	White-browed Treecreeper	PASSERIFORMES	Climacteridae	1	2
Climacteris erythrops	Red-browed Treecreeper	PASSERIFORMES	Climacteridae	3	3
Climacteris melanura	Black-tailed Treecreeper	PASSERIFORMES	Climacteridae	3	2
Climacteris picumnus	Brown Treecreeper	PASSERIFORMES	Climacteridae	3	3
Climacteris rufa	Rufous Treecreeper	PASSERIFORMES	Climacteridae	2	3

Colluricincla boweri	Bower's Shrike-thrush	PASSERIFORMES	Pachycephalidae	2	3
Colluricincla harmonica	Grey Shrike-thrush	PASSERIFORMES	Pachycephalidae	2	3
Columba leucomela	White-headed Pigeon	COLUMBIFORMES	Columbidae	2	3
Colluricincla megarhyncha	Little Shrike-thrush	PASSERIFORMES	Pachycephalidae	3	3
Colluricincla woodwardi	Sandstone Shrike-thrush	PASSERIFORMES	Pachycephalidae	2	3
Conopophila albogularis	Rufous-banded Honeyeater	PASSERIFORMES	Meliphagidae	3	3
Conopophila rufogularis	Rufous-throated Honeyeater	PASSERIFORMES	Meliphagidae	3	3
Conopophila whitei	Grey Honeyeater	PASSERIFORMES	Meliphagidae	3	4
Cormobates leucophaea	White-throated Treecreeper	PASSERIFORMES	Climacteridae	3	3
Coracina lineata	Barred Cuckoo-shrike	PASSERIFORMES	Campephagidae	2	3
Coracina maxima	Ground Cuckoo-shrike	PASSERIFORMES	Campephagidae	1	1
Coracina novaehollandiae	Black-faced Cuckoo-shrike	PASSERIFORMES	Campephagidae	3	3
Coracina papuensis	White-bellied Cuckoo-shrike	PASSERIFORMES	Campephagidae	1	1
Coracina tenuirostris	Cicadabird	PASSERIFORMES	Campephagidae	2	3
Corcorax melanorhamphos	White-winged Chough	PASSERIFORMES	Corcoracidae	3	3
Corvus bennetti	Little Crow	PASSERIFORMES	Corvidae	2	3
Corvus coronoides	Australian Raven	PASSERIFORMES	Corvidae	3	3
Corvus mellori	Little Raven	PASSERIFORMES	Corvidae	3	3
Corvus orru	Torresian Crow	PASSERIFORMES	Corvidae	3	3
Corvus tasmanicus	Forest Raven	PASSERIFORMES	Corvidae	2	2
Coturnix pectoralis	Stubble Quail	GALLIFORMES	Phasianidae	3	3
Coturnix ypsilophora	Brown Quail	GALLIFORMES	Phasianidae	3	3
Cracticus mentalis	Black-backed Butcherbird	PASSERIFORMES	Artamidae	2	2
Cracticus nigrogularis	Pied Butcherbird	PASSERIFORMES	Artamidae	1	3
Cracticus quoyi	Black Butcherbird	PASSERIFORMES	Artamidae	3	3
Cracticus tibicen	Australian Magpie	PASSERIFORMES	Artamidae	3	3
Cracticus torquatus	Grey Butcherbird	PASSERIFORMES	Artamidae	2	3
Cyclopsitta diophthalma	Double-eyed Fig-Parrot	PSITTACIFORMES	Psittacidae	0	1
Cygnus atratus	Black Swan	ANSERIFORMES	Anatidae	1	0
Dacelo leachii	Blue-winged Kookaburra	CORACIIFORMES	Halcyonidae	3	3
Dacelo novaeguineae	Laughing Kookaburra	CORACIIFORMES	Halcyonidae	3	3
Daphoenositta chrysoptera	Varied Sittella	PASSERIFORMES	Neosittidae	3	3
Dasyornis brachypterus	Eastern Bristlebird	PASSERIFORMES	Dasyornithidae	2	3
Dasyornis broadbenti	Rufous Bristlebird	PASSERIFORMES	Dasyornithidae	1	3
Dasyornis longirostris	Western Bristlebird	PASSERIFORMES	Dasyornithidae	1	1
Dendrocygna arcuata	Wandering Whistling-Duck	ANSERIFORMES	Anatidae	1	2
Dendrocygna eytoni	Plumed Whistling-Duck	ANSERIFORMES	Anatidae	3	2
Dicrurus bracteatus	Spangled Drongo	PASSERIFORMES	Dicruridae	3	2
Dicaeum hirundinaceum	Mistletoebird	PASSERIFORMES	Nectariniidae	3	3
Dromaius novaehollandiae	Emu	CASUARIIFORMES	Casuariidae	2	0
Drymodes brunneopygia	Southern Scrub-robin	PASSERIFORMES	Petroicidae	2	3
Drymodes superciliaris	Northern Scrub-robin	PASSERIFORMES	Petroicidae	0	4
Ducula bicolor	Pied Imperial-Pigeon	COLUMBIFORMES	Columbidae	3	3
Eclectus roratus	Eclectus Parrot	PSITTACIFORMES	Psittacidae	3	3
Ardea alba	Great Egret	CICONIIFORMES	Ardeidae	1	3
Egretta garzetta	Little Egret	CICONIIFORMES	Ardeidae	1	2

Egretta novaehollandiae	White-faced Heron	CICONIIFORMES	Ardeidae	3	3
Egretta picata	Pied Heron	CICONIIFORMES	Ardeidae	3	3
Egretta sacra	Eastern Reef Egret	CICONIIFORMES	Ardeidae	3	2
Elanus axillaris	Black-shouldered Kite	ACCIPITRIFORMES	Accipitridae	3	3
Elanus scriptus	Letter-winged Kite	ACCIPITRIFORMES	Accipitridae	3	2
Elseyornis melanops	Black-fronted Dotterel	CHARADRIIFORMES	Charadriidae	3	3
Emblema pictum	Painted Finch	PASSERIFORMES	Estrildidae	3	3
Entomyzon cyanotis	Blue-faced Honeyeater	PASSERIFORMES	Meliphagidae	3	3
Eolophus roseicapillus	Galah	PSITTACIFORMES	Cacatuidae	3	3
Eopsaltria australis	Eastern Yellow Robin	PASSERIFORMES	Petroicidae	3	3
Eopsaltria georgiana	White-breasted Robin	PASSERIFORMES	Petroicidae	2	2
Eopsaltria griseogularis	Western Yellow Robin	PASSERIFORMES	Petroicidae	2	3
Ephippiorhynchus asiaticus	Black-necked Stork	CICONIIFORMES	Ciconiidae	1	0
Epthianura albifrons	White-fronted Chat	PASSERIFORMES	Meliphagidae	3	3
Epthianura aurifrons	Orange Chat	PASSERIFORMES	Meliphagidae	3	3
Epthianura crocea	Yellow Chat	PASSERIFORMES	Meliphagidae	2	2
Epthianura tricolor	Crimson Chat	PASSERIFORMES	Meliphagidae	3	3
Eremiornis carteri	Spinifexbird	PASSERIFORMES	Megaluridae	2	3
Erythrogonys cinctus	Red-kneed Dotterel	CHARADRIIFORMES	Charadriidae	3	3
Erythrura gouldiae	Gouldian Finch	PASSERIFORMES	Estrildidae	3	3
Erythrotriorchis radiatus	Red Goshawk	ACCIPITRIFORMES	Accipitridae	3	3
Erythrura trichroa	Blue-faced Parrot-Finch	PASSERIFORMES	Estrildidae	1	1
Esacus magnirostris	Beach Stone-curlew	CHARADRIIFORMES	Burhinidae	1	1
Eudynamys scolopaceus	Asian Koel	CUCULIFORMES	Cuculidae	3	3
Eulabeornis castaneoventris	Chestnut Rail	GRUIFORMES	Rallidae	3	1
Eurostopodus argus	Spotted Nightjar	CAPRIMULGIFORMES	Eurostopodidae	3	3
Eurostopodus mystacalis	White-throated Nightjar	CAPRIMULGIFORMES	Eurostopodidae	2	2
Eurystomus orientalis	Dollarbird	CORACIIFORMES	Coraciidae	3	3
Excalfactoria chinensis	King Quail	GALLIFORMES	Phasianidae	3	3
Falco berigora	Brown Falcon	FALCONIFORMES	Falconidae	3	3
Falco cenchroides	Nankeen Kestrel	FALCONIFORMES	Falconidae	3	3
Falcunculus frontatus	Crested Shrike-tit	PASSERIFORMES	Pachycephalidae	3	3
Falco hypoleucos	Grey Falcon	FALCONIFORMES	Falconidae	1	3
Falco longipennis	Australian Hobby	FALCONIFORMES	Falconidae	3	3
Falco peregrinus	Peregrine Falcon	FALCONIFORMES	Falconidae	3	3
Falco subniger	Black Falcon	FALCONIFORMES	Falconidae	2	3
Fulica atra	Eurasian Coot	GRUIFORMES	Rallidae	3	3
Gallirallus philippensis	Buff-banded Rail	GRUIFORMES	Rallidae	3	3
Gallinula tenebrosa	Dusky Moorhen	GRUIFORMES	Rallidae	3	3
Gelochelidon nilotica	Gull-billed Tern	CHARADRIIFORMES	Laridae	1	3
Geopelia cuneata	Diamond Dove	COLUMBIFORMES	Columbidae	3	3
Geoffroyus geoffroyi	Red-cheeked Parrot	PSITTACIFORMES	Psittacidae	2	2
Geopelia humeralis	Bar-shouldered Dove	COLUMBIFORMES	Columbidae	3	3
Geophaps plumifera	Spinifex Pigeon	COLUMBIFORMES	Columbidae	3	3
Geophaps scripta	Squatter Pigeon	COLUMBIFORMES	Columbidae	2	2
Geophaps smithii	Partridge Pigeon	COLUMBIFORMES	Columbidae	2	3

Geopelia striata	Peaceful Dove	COLUMBIFORMES	Columbidae	3	3
Gerygone albogularis	White-throated Gerygone	PASSERIFORMES	Acanthizidae	3	2
Gerygone chloronota	Green-backed Gerygone	PASSERIFORMES	Acanthizidae	1	3
Gerygone fusca	Western Gerygone	PASSERIFORMES	Acanthizidae	2	2
Gerygone levigaster	Mangrove Gerygone	PASSERIFORMES	Acanthizidae	3	1
Gerygone magnirostris	Large-billed Gerygone	PASSERIFORMES	Acanthizidae	2	2
Gerygone mouki	Brown Gerygone	PASSERIFORMES	Acanthizidae	2	2
Gerygone palpebrosa	Fairy Gerygone	PASSERIFORMES	Acanthizidae	1	3
Gerygone tenebrosa	Dusky Gerygone	PASSERIFORMES	Acanthizidae	3	3
Glossopsitta concinna	Musk Lorikeet	PSITTACIFORMES	Psittacidae	3	3
Glossopsitta porphyrocephala	Purple-crowned Lorikeet	PSITTACIFORMES	Psittacidae	3	3
Glossopsitta pusilla	Little Lorikeet	PSITTACIFORMES	Psittacidae	2	3
Glycichaera fallax	Green-backed Honeyeater	PASSERIFORMES	Meliphagidae	2	2
Glyciphila melanops	Tawny-crowned Honeyeater	PASSERIFORMES	Meliphagidae	3	3
Grallina cyanoleuca	Magpie-lark	PASSERIFORMES	Monarchidae	3	3
Grantiella picta	Painted Honeyeater	PASSERIFORMES	Meliphagidae	3	3
Grus antigone	Sarus Crane	GRUIFORMES	Gruidae	3	2
Grus rubicunda	Brolga	GRUIFORMES	Gruidae	2	3
Haematopus fuliginosus	Sooty Oystercatcher	CHARADRIIFORMES	Haematopodidae	1	3
Haematopus longirostris	Australian Pied Oystercatcher	CHARADRIIFORMES	Haematopodidae	3	2
Haliastur indus	Brahminy Kite	ACCIPITRIFORMES	Accipitridae	2	1
Haliaeetus leucogaster	White-bellied Sea-Eagle	ACCIPITRIFORMES	Accipitridae	3	1
Haliastur sphenurus	Whistling Kite	ACCIPITRIFORMES	Accipitridae	2	3
Hamirostra melanosternon	Black-breasted Buzzard	ACCIPITRIFORMES	Accipitridae	2	2
Heteromyias cinereifrons	Grey-headed Robin	PASSERIFORMES	Petroicidae	3	3
Heteromunia pectoralis	Pictorella Mannikin	PASSERIFORMES	Estrildidae	3	3
Hieraaetus morphnoides	Little Eagle	ACCIPITRIFORMES	Accipitridae	3	2
Himantopus himantopus	Black-winged Stilt	CHARADRIIFORMES	Recurvirostridae	3	1
Hirundo neoxena	Welcome Swallow	PASSERIFORMES	Hirundinidae	3	3
Hydroprogne caspia	Caspian Tern	CHARADRIIFORMES	Laridae	1	2
Hylacola cauta	Shy Heathwren	PASSERIFORMES	Acanthizidae	2	3
Hylacola pyrrhopygia	Chestnut-rumped Heathwren	PASSERIFORMES	Acanthizidae	3	3
Irediparra gallinacea	Comb-crested Jacana	CHARADRIIFORMES	Jacanidae	3	1
Ixobrychus dubius	Australian Little Bittern	CICONIIFORMES	Ardeidae	2	3
Ixobrychus flavicollis	Black Bittern	CICONIIFORMES	Ardeidae	1	2
Lalage leucomela	Varied Triller	PASSERIFORMES	Campephagidae	3	3
Lalage sueurii	White-winged Triller	PASSERIFORMES	Campephagidae	2	3
Larus dominicanus	Kelp Gull	CHARADRIIFORMES	Laridae	3	3
Larus pacificus	Pacific Gull	CHARADRIIFORMES	Laridae	1	3
Lathamus discolor	Swift Parrot	PSITTACIFORMES	Psittacidae	3	3
Leipoa ocellata	Malleefowl	GALLIFORMES	Megapodiidae	1	1
Leucosarcia picata	Wonga Pigeon	COLUMBIFORMES	Columbidae	2	2
Lewinia pectoralis	Lewin's Rail	GRUIFORMES	Rallidae	3	3
Lichenostomus chrysops	Yellow-faced Honeyeater	PASSERIFORMES	Meliphagidae	3	3
Lichenostomus cratitius	Purple-gaped Honeyeater	PASSERIFORMES	Meliphagidae	3	3

Lichenostomus fasciogularis	Mangrove Honeyeater	PASSERIFORMES	Meliphagidae	1	1
Lichenostomus flavescens	Yellow-tinted Honeyeater	PASSERIFORMES	Meliphagidae	3	3
Lichenostomus flavicollis	Yellow-throated Honeyeater	PASSERIFORMES	Meliphagidae	3	3
Lichenostomus flavus	Yellow Honeyeater	PASSERIFORMES	Meliphagidae	3	2
Lichenostomus frenatus	Bridled Honeyeater	PASSERIFORMES	Meliphagidae	3	3
Lichenostomus fuscus	Fuscous Honeyeater	PASSERIFORMES	Meliphagidae	3	3
Lichenostomus hindwoodi	Eungella Honeyeater	PASSERIFORMES	Meliphagidae	2	1
Lichmera indistincta	Brown Honeyeater	PASSERIFORMES	Meliphagidae	3	3
Lichenostomus keartlandi	Grey-headed Honeyeater	PASSERIFORMES	Meliphagidae	2	1
Lichenostomus leucotis	White-eared Honeyeater	PASSERIFORMES	Meliphagidae	3	3
Lichenostomus melanops	Yellow-tufted Honeyeater	PASSERIFORMES	Meliphagidae	3	3
Lichenostomus ornatus	Yellow-plumed Honeyeater	PASSERIFORMES	Meliphagidae	3	3
Lichenostomus penicillatus	White-plumed Honeyeater	PASSERIFORMES	Meliphagidae	3	3
Lichenostomus plumulus	Grey-fronted Honeyeater	PASSERIFORMES	Meliphagidae	2	3
Lichenostomus unicolor	White-gaped Honeyeater	PASSERIFORMES	Meliphagidae	3	3
Lichenostomus versicolor	Varied Honeyeater	PASSERIFORMES	Meliphagidae	3	4
Lichenostomus virescens	Singing Honeyeater	PASSERIFORMES	Meliphagidae	3	3
Lonchura castaneothorax	Chestnut-breasted Mannikin	PASSERIFORMES	Estrildidae	3	3
Lonchura flaviprymna	Yellow-rumped Mannikin	PASSERIFORMES	Estrildidae	1	2
Lopholaimus antarcticus	Topknot Pigeon	COLUMBIFORMES	Columbidae	3	3
Lophoictinia isura	Square-tailed Kite	ACCIPITRIFORMES	Accipitridae	1	3
Lophochroa leadbeateri	Major Mitchell's Cockatoo	PSITTACIFORMES	Cacatuidae	3	3
Macropygia amboinensis	Brown Cuckoo-Dove	COLUMBIFORMES	Columbidae	1	3
Machaerirhynchus flaviventer	Yellow-breasted Boatbill	PASSERIFORMES	Monarchidae	1	3
Malurus amabilis	Lovely Fairy-wren	PASSERIFORMES	Maluridae	2	3
Malurus coronatus	Purple-crowned Fairy-wren	PASSERIFORMES	Maluridae	3	2
Malurus cyaneus	Superb Fairy-wren	PASSERIFORMES	Maluridae	3	3
Malurus elegans	Red-winged Fairy-wren	PASSERIFORMES	Maluridae	2	3
Malurus lamberti	Variegated Fairy-wren	PASSERIFORMES	Maluridae	2	3
Malurus leucopterus	White-winged Fairy-wren	PASSERIFORMES	Maluridae	3	3
Malurus melanocephalus	Red-backed Fairy-wren	PASSERIFORMES	Maluridae	3	3
Malacorhynchus membranaceus	Pink-eared Duck	ANSERIFORMES	Anatidae	2	3
Malurus pulcherrimus	Blue-breasted Fairy-wren	PASSERIFORMES	Maluridae	2	3
Malurus splendens	Splendid Fairy-wren	PASSERIFORMES	Maluridae	2	3
Manorina flavigula	Yellow-throated Miner	PASSERIFORMES	Meliphagidae	3	3
Manorina melanocephala	Noisy Miner	PASSERIFORMES	Meliphagidae	3	3
Manorina melanophrys	Bell Miner	PASSERIFORMES	Meliphagidae	3	3
Manorina melanotis	Black-eared Miner	PASSERIFORMES	Meliphagidae	1	2
Megalurus gramineus	Little Grassbird	PASSERIFORMES	Megaluridae	2	3
Megapodius reinwardt	Orange-footed Scrubfowl	GALLIFORMES	Megapodiidae	2	3
Megalurus timoriensis	Tawny Grassbird	PASSERIFORMES	Megaluridae	1	1
Melithreptus affinis	Black-headed Honeyeater	PASSERIFORMES	Meliphagidae	3	1
Meliphaga albilineata	White-lined Honeyeater	PASSERIFORMES	Meliphagidae	1	1
Melithreptus albogularis	White-throated Honeyeater	PASSERIFORMES	Meliphagidae	1	3
Melithreptus brevirostris	Brown-headed Honeyeater	PASSERIFORMES	Meliphagidae	2	3
Melanodryas cucullata	Hooded Robin	PASSERIFORMES	Petroicidae	3	3

Meliphaga gracilis	Graceful Honeyeater	PASSERIFORMES	Meliphagidae	1	3
Melithreptus gularis	Black-chinned Honeyeater	PASSERIFORMES	Meliphagidae	3	3
Meliphaga lewinii	Lewin's Honeyeater	PASSERIFORMES	Meliphagidae	3	3
Melithreptus lunatus	White-naped Honeyeater	PASSERIFORMES	Meliphagidae	3	3
Meliphaga notata	Yellow-spotted Honeyeater	PASSERIFORMES	Meliphagidae	2	3
Melopsittacus undulatus	Budgerigar	PSITTACIFORMES	Psittacidae	3	3
Melithreptus validirostris	Strong-billed Honeyeater	PASSERIFORMES	Meliphagidae	1	3
Melanodryas vittata	Dusky Robin	PASSERIFORMES	Petroicidae	2	2
Menura alberti	Albert's Lyrebird	PASSERIFORMES	Menuridae	2	2
Menura novaehollandiae	Superb Lyrebird	PASSERIFORMES	Menuridae	3	3
Merops ornatus	Rainbow Bee-eater	CORACIIFORMES	Meropidae	3	3
Microeca fascinans	Jacky Winter	PASSERIFORMES	Petroicidae	1	2
Microeca flavigaster	Lemon-bellied Flycatcher	PASSERIFORMES	Petroicidae	3	3
Microeca griseoceps	Yellow-legged Flycatcher	PASSERIFORMES	Petroicidae	1	2
Microcarbo melanoleucos	Little Pied Cormorant	PHALACROCORACIFORMES	Phalacrocoracidae	3	3
Milvus migrans	Black Kite	ACCIPITRIFORMES	Accipitridae	3	3
Mirafra javanica	Horsfield's Bushlark	PASSERIFORMES	Alaudidae	2	3
Monarcha frater	Black-winged Monarch	PASSERIFORMES	Monarchidae	3	2
Monarcha melanopsis	Black-faced Monarch	PASSERIFORMES	Monarchidae	2	3
Myiagra alecto	Shining Flycatcher	PASSERIFORMES	Monarchidae	3	3
Myiagra cyanoleuca	Satin Flycatcher	PASSERIFORMES	Monarchidae	3	3
Myiagra inquieta	Restless Flycatcher	PASSERIFORMES	Monarchidae	1	3
Myiagra rubecula	Leaden Flycatcher	PASSERIFORMES	Monarchidae	2	3
Myiagra ruficollis	Broad-billed Flycatcher	PASSERIFORMES	Monarchidae	1	2
Myzomela erythrocephala	Red-headed Honeyeater	PASSERIFORMES	Meliphagidae	3	3
Myzomela obscura	Dusky Honeyeater	PASSERIFORMES	Meliphagidae	3	3
Myzomela sanguinolenta	Scarlet Honeyeater	PASSERIFORMES	Meliphagidae	1	3
Nectarinia jugularis	Olive-backed Sunbird	PASSERIFORMES	Nectariniidae	3	3
Neopsephotus bourkii	Bourke's Parrot	PSITTACIFORMES	Psittacidae	2	2
Neophema chrysogaster	Orange-bellied Parrot	PSITTACIFORMES	Psittacidae	0	2
Neophema chrysostoma	Blue-winged Parrot	PSITTACIFORMES	Psittacidae	3	3
Neophema elegans	Elegant Parrot	PSITTACIFORMES	Psittacidae	1	3
Neochmia modesta	Plum-headed Finch	PASSERIFORMES	Estrildidae	2	2
Neophema petrophila	Rock Parrot	PSITTACIFORMES	Psittacidae	2	2
Neochmia phaeton	Crimson Finch	PASSERIFORMES	Estrildidae	2	3
Neophema pulchella	Turquoise Parrot	PSITTACIFORMES	Psittacidae	3	1
Neochmia ruficauda	Star Finch	PASSERIFORMES	Estrildidae	2	1
Neophema splendida	Scarlet-chested Parrot	PSITTACIFORMES	Psittacidae	3	3
Neochmia temporalis	Red-browed Finch	PASSERIFORMES	Estrildidae	2	3
Nettapus coromandelianus	Cotton Pygmy-goose	ANSERIFORMES	Anatidae	2	3
Nettapus pulchellus	Green Pygmy-goose	ANSERIFORMES	Anatidae	3	3
Ninox connivens	Barking Owl	STRIGIFORMES	Strigidae	3	1
Ninox novaeseelandiae	Southern Boobook	STRIGIFORMES	Strigidae	3	3
Ninox rufa	Rufous Owl	STRIGIFORMES	Strigidae	3	3
Ninox strenua	Powerful Owl	STRIGIFORMES	Strigidae	3	3
Northiella haematogaster	Blue Bonnet	PSITTACIFORMES	Psittacidae	3	3

Nycticorax caledonicus	Nankeen Night-Heron	CICONIIFORMES	Ardeidae	1	3
Nymphicus hollandicus	Cockatiel	PSITTACIFORMES	Cacatuidae	3	3
Ocyphaps lophotes	Crested Pigeon	COLUMBIFORMES	Columbidae	3	3
Oreoica gutturalis	Crested Bellbird	PASSERIFORMES	Pachycephalidae	3	3
Oreoscopus gutturalis	Fernwren	PASSERIFORMES	Acanthizidae	1	2
Oriolus flavocinctus	Yellow Oriole	PASSERIFORMES	Oriolidae	3	3
Oriolus sagittatus	Olive-backed Oriole	PASSERIFORMES	Oriolidae	3	3
Origma solitaria	Rockwarbler	PASSERIFORMES	Acanthizidae	3	1
Orthonyx spaldingii	Chowchilla	PASSERIFORMES	Orthonychidae	2	2
Orthonyx temminckii	Australian Logrunner	PASSERIFORMES	Orthonychidae	3	3
Oxyura australis	Blue-billed Duck	ANSERIFORMES	Anatidae	3	3
Pachycephala inornata	Gilbert's Whistler	PASSERIFORMES	Pachycephalidae	3	3
Pachycephala lanioides	White-breasted Whistler	PASSERIFORMES	Pachycephalidae	3	3
Pachycephala melanura	Mangrove Golden Whistler	PASSERIFORMES	Pachycephalidae	2	3
Pachycephala olivacea	Olive Whistler	PASSERIFORMES	Pachycephalidae	3	3
Pachycephala pectoralis	Golden Whistler	PASSERIFORMES	Pachycephalidae	2	3
Pachycephala rufiventris	Rufous Whistler	PASSERIFORMES	Pachycephalidae	3	3
Pachycephala rufogularis	Red-lored Whistler	PASSERIFORMES	Pachycephalidae	1	3
Pachycephala simplex	Grey Whistler	PASSERIFORMES	Pachycephalidae	2	1
Pandion cristatus	Eastern Osprey	ACCIPITRIFORMES	Accipitridae	1	2
Pardalotus punctatus	Spotted Pardalote	PASSERIFORMES	Pardalotidae	3	3
Pardalotus quadragintus	Forty-spotted Pardalote	PASSERIFORMES	Pardalotidae	2	2
Pardalotus rubricatus	Red-browed Pardalote	PASSERIFORMES	Pardalotidae	3	3
Pardalotus striatus	Striated Pardalote	PASSERIFORMES	Pardalotidae	3	3
Pedionomus torquatus	Plains-wanderer	CHARADRIIFORMES	Pedionomidae	3	3
Pelecanus conspicillatus	Australian Pelican	CICONIIFORMES	Pelecanidae	0	3
Peneonanthe pulverulenta	Mangrove Robin	PASSERIFORMES	Petroicidae	3	2
Petrophassa albipennis	White-quilled Rock-Pigeon	COLUMBIFORMES	Columbidae	1	2
Petrochelidon ariel	Fairy Martin	PASSERIFORMES	Hirundinidae	2	3
Petroica boodang	Scarlet Robin	PASSERIFORMES	Petroicidae	3	3
Petroica goodenovii	Red-capped Robin	PASSERIFORMES	Petroicidae	3	3
Petrochelidon nigricans	Tree Martin	PASSERIFORMES	Hirundinidae	2	3
Petroica phoenicea	Flame Robin	PASSERIFORMES	Petroicidae	3	3
Petroica rodinogaster	Pink Robin	PASSERIFORMES	Petroicidae	1	3
Petroica rosea	Rose Robin	PASSERIFORMES	Petroicidae	3	3
Petrophassa rufipennis	Chestnut-quilled Rock-Pigeon	COLUMBIFORMES	Columbidae	3	3
Pezoporus wallicus	Ground Parrot	PSITTACIFORMES	Psittacidae	2	2
Phalacrocorax carbo	Great Cormorant	PHALACROCORACIFORMES	Phalacrocoracidae	3	3
Phaps chalcoptera	Common Bronzewing	COLUMBIFORMES	Columbidae	3	3
Phaps elegans	Brush Bronzewing	COLUMBIFORMES	Columbidae	3	3
Phalacrocorax fuscescens	Black-faced Cormorant	PHALACROCORACIFORMES	Phalacrocoracidae	3	2
Phaps histrionica	Flock Bronzewing	COLUMBIFORMES	Columbidae	1	3
Phalacrocorax sulcirostris	Little Black Cormorant	PHALACROCORACIFORMES	Phalacrocoracidae	2	3
Phalacrocorax varius	Pied Cormorant	PHALACROCORACIFORMES	Phalacrocoracidae	3	3
Philemon argenticeps	Silver-crowned Friarbird	PASSERIFORMES	Meliphagidae	2	1

Philemon buceroides	Helmeted Friarbird	PASSERIFORMES	Meliphagidae	3	3
Philemon citreogularis	Little Friarbird	PASSERIFORMES	Meliphagidae	1	3
Philemon corniculatus	Noisy Friarbird	PASSERIFORMES	Meliphagidae	1	3
Phonygammus keraudrenii	Trumpet Manucode	PASSERIFORMES	Paradisaeidae	1	3
Phylidonyris niger	White-cheeked Honeyeater	PASSERIFORMES	Meliphagidae	3	2
Phylidonyris novaehollandiae	New Holland Honeyeater	PASSERIFORMES	Meliphagidae	3	3
Phylidonyris pyrrhopterus	Crescent Honeyeater	PASSERIFORMES	Meliphagidae	3	3
Pitta erythrogaster	Red-bellied Pitta	PASSERIFORMES	Pittidae	0	3
Pitta iris	Rainbow Pitta	PASSERIFORMES	Pittidae	3	3
Pitta versicolor	Noisy Pitta	PASSERIFORMES	Pittidae	2	1
Platycercus adscitus	Pale-headed Rosella	PSITTACIFORMES	Psittacidae	3	3
Platycercus caledonicus	Green Rosella	PSITTACIFORMES	Psittacidae	3	2
Platycercus elegans	Crimson Rosella	PSITTACIFORMES	Psittacidae	3	3
Platycercus eximius	Eastern Rosella	PSITTACIFORMES	Psittacidae	3	3
Platalea flavipes	Yellow-billed Spoonbill	CICONIIFORMES	Threskiornithidae	1	1
Platycercus icterotis	Western Rosella	PSITTACIFORMES	Psittacidae	3	3
Platalea regia	Royal Spoonbill	CICONIIFORMES	Threskiornithidae	2	0
Platycercus venustus	Northern Rosella	PSITTACIFORMES	Psittacidae	3	3
Plegadis falcinellus	Glossy Ibis	CICONIIFORMES	Threskiornithidae	1	1
Plectorhyncha lanceolata	Striped Honeyeater	PASSERIFORMES	Meliphagidae	3	3
Podiceps cristatus	Great Crested Grebe	PODICIPEDIFORMES	Podicipedidae	3	3
Podargus ocellatus	Marbled Frogmouth	CAPRIMULGIFORMES	Podargidae	1	2
Podargus papuensis	Papuan Frogmouth	CAPRIMULGIFORMES	Podargidae	2	3
Podargus strigoides	Tawny Frogmouth	CAPRIMULGIFORMES	Podargidae	3	3
Poephila acuticauda	Long-tailed Finch	PASSERIFORMES	Estrildidae	3	3
Poecilodryas cerviniventris	Buff-sided Robin	PASSERIFORMES	Petroicidae	2	1
Poephila cincta	Black-throated Finch	PASSERIFORMES	Estrildidae	3	3
Poephila personata	Masked Finch	PASSERIFORMES	Estrildidae	1	1
Poecilodryas superciliosa	White-browed Robin	PASSERIFORMES	Petroicidae	2	2
Polytelis alexandrae	Princess Parrot	PSITTACIFORMES	Psittacidae	1	4
Polytelis anthopeplus	Regent Parrot	PSITTACIFORMES	Psittacidae	3	2
Poliocephalus poliocephalus	Hoary-headed Grebe	PODICIPEDIFORMES	Podicipedidae	2	3
Polytelis swainsonii	Superb Parrot	PSITTACIFORMES	Psittacidae	2	3
Pomatostomus halli	Hall's Babbler	PASSERIFORMES	Pomatostomidae	2	2
Pomatostomus ruficeps	Chestnut-crowned Babbler	PASSERIFORMES	Pomatostomidae	2	2
Pomatostomus superciliosus	White-browed Babbler	PASSERIFORMES	Pomatostomidae	3	3
Pomatostomus temporalis	Grey-crowned Babbler	PASSERIFORMES	Pomatostomidae	3	3
Porzana fluminea	Australian Spotted Crake	GRUIFORMES	Rallidae	3	3
Porphyrio porphyrio	Purple Swamphen	GRUIFORMES	Rallidae	3	3
Porzana pusilla	Baillon's Crake	GRUIFORMES	Rallidae	2	3
Porzana tabuensis	Spotless Crake	GRUIFORMES	Rallidae	3	3
Probosciger aterrimus	Palm Cockatoo	PSITTACIFORMES	Cacatuidae	1	2
Psephotus chrysopterygius	Golden-shouldered Parrot	PSITTACIFORMES	Psittacidae	1	1
Psephotus dissimilis	Hooded Parrot	PSITTACIFORMES	Psittacidae	1	3

Psephotus haematonotus	Red-rumped Parrot	PSITTACIFORMES	Psittacidae	3	3
Psephotus varius	Mulga Parrot	PSITTACIFORMES	Psittacidae	3	3
Psitteuteles versicolor	Varied Lorikeet	PSITTACIFORMES	Psittacidae	3	3
Psophodes cristatus	Chirruping Wedgebill	PASSERIFORMES	Psophodidae	2	1
Psophodes nigrogularis	Western Whipbird	PASSERIFORMES	Psophodidae	0	2
Psophodes occidentalis	Chiming Wedgebill	PASSERIFORMES	Psophodidae	1	1
Psophodes olivaceus	Eastern Whipbird	PASSERIFORMES	Psophodidae	3	3
Ptilonorhynchus cerviniventris	Fawn-breasted Bowerbird	PASSERIFORMES	Ptilonorhynchidae	2	1
Ptilinopus cinctus	Banded Fruit-Dove	COLUMBIFORMES	Columbidae	3	3
Ptilonorhynchus guttatus	Western Bowerbird	PASSERIFORMES	Ptilonorhynchidae	3	3
Ptilonorhynchus maculatus	Spotted Bowerbird	PASSERIFORMES	Ptilonorhynchidae	3	3
Ptiloris magnificus	Magnificent Riflebird	PASSERIFORMES	Paradisaeidae	1	3
Ptilonorhynchus nuchalis	Great Bowerbird	PASSERIFORMES	Ptilonorhynchidae	2	3
Ptiloris paradiseus	Paradise Riflebird	PASSERIFORMES	Paradisaeidae	2	2
Ptilinopus regina	Rose-crowned Fruit-Dove	COLUMBIFORMES	Columbidae	3	3
Ptilinopus superbus	Superb Fruit-Dove	COLUMBIFORMES	Columbidae	2	3
Ptiloris victoriae	Victoria's Riflebird	PASSERIFORMES	Paradisaeidae	3	3
Ptilonorhynchus violaceus	Satin Bowerbird	PASSERIFORMES	Ptilonorhynchidae	3	3
Ptilinopus magnificus	Wompoo Fruit-Dove	COLUMBIFORMES	Columbidae	2	3
Purnella albifrons	White-fronted Honeyeater	PASSERIFORMES	Meliphagidae	3	3
Purpureicephalus spurius	Red-capped Parrot	PSITTACIFORMES	Psittacidae	3	3
Pycnoptilus floccosus	Pilotbird	PASSERIFORMES	Acanthizidae	1	1
Pyrrholaemus brunneus	Redthroat	PASSERIFORMES	Acanthizidae	3	3
Rallina tricolor	Red-necked Crake	GRUIFORMES	Rallidae	2	3
Ramsayornis fasciatus	Bar-breasted Honeyeater	PASSERIFORMES	Meliphagidae	1	3
Ramsayornis modestus	Brown-backed Honeyeater	PASSERIFORMES	Meliphagidae	5	1
Recurvirostra novaehollandiae	Red-necked Avocet	CHARADRIIFORMES	Recurvirostridae	1	3
Rhipidura albiscapa	Grey Fantail	PASSERIFORMES	Rhipiduridae	3	3
Rhipidura dryas	Arafura Fantail	PASSERIFORMES	Rhipiduridae	3	6
Rhipidura leucophrys	Willie Wagtail	PASSERIFORMES	Rhipiduridae	3	3
Rhipidura phasiana	Mangrove Grey Fantail	PASSERIFORMES	Rhipiduridae	3	2
Rhipidura rufifrons	Rufous Fantail	PASSERIFORMES	Rhipiduridae	2	3
Rhipidura rufiventris	Northern Fantail	PASSERIFORMES	Rhipiduridae	3	3
Rostratula australis	Australian Painted Snipe	CHARADRIIFORMES	Rostratulidae	3	3
Scenopoeetes dentirostris	Tooth-billed Bowerbird	PASSERIFORMES	Ptilonorhynchidae	3	3
Scythrops novaehollandiae	Channel-billed Cuckoo	CUCULIFORMES	Cuculidae	2	2
Sericornis beccarii	Tropical Scrubwren	PASSERIFORMES	Acanthizidae	1	2
Sericulus chrysocephalus	Regent Bowerbird	PASSERIFORMES	Ptilonorhynchidae	6	6
Sericornis citreogularis	Yellow-throated Scrubwren	PASSERIFORMES	Acanthizidae	3	1
Sericornis frontalis	White-browed Scrubwren	PASSERIFORMES	Acanthizidae	3	3
Sericornis humilis	Tasmanian Scrubwren	PASSERIFORMES	Acanthizidae	3	3
Sericornis keri	Atherton Scrubwren	PASSERIFORMES	Acanthizidae	2	2
Sericornis magnirostra	Large-billed Scrubwren	PASSERIFORMES	Acanthizidae	3	3
Smicrornis brevirostris	Weebill	PASSERIFORMES	Acanthizidae	3	3

Sphecotheres vieilloti	Australasian Figbird	PASSERIFORMES	Oriolidae	3	3
Stagonopleura bella	Beautiful Firetail	PASSERIFORMES	Estrildidae	1	3
Stagonopleura guttata	Diamond Firetail	PASSERIFORMES	Estrildidae	3	3
Stagonopleura oculata	Red-eared Firetail	PASSERIFORMES	Estrildidae	1	2
Sternula albifrons	Little Tern	CHARADRIIFORMES	Laridae	2	1
Sternula nereis	Fairy Tern	CHARADRIIFORMES	Laridae	2	2
Sterna striata	White-fronted Tern	CHARADRIIFORMES	Laridae	0	2
Sterna sumatrana	Black-naped Tern	CHARADRIIFORMES	Laridae	2	3
Stiltia isabella	Australian Pratincole	CHARADRIIFORMES	Glareolidae	1	1
Stipiturus malachurus	Southern Emu-wren	PASSERIFORMES	Maluridae	2	3
Stipiturus mallee	Mallee Emu-wren	PASSERIFORMES	Maluridae	3	3
Stictonetta naevosa	Freckled Duck	ANSERIFORMES	Anatidae	3	3
Stipiturus ruficeps	Rufous-crowned Emu-wren	PASSERIFORMES	Maluridae	3	3
Struthidea cinerea	Apostlebird	PASSERIFORMES	Corcoracidae	3	3
Strepera fuliginosa	Black Currawong	PASSERIFORMES	Artamidae	3	1
Strepera graculina	Pied Currawong	PASSERIFORMES	Artamidae	2	3
Strepera versicolor	Grey Currawong	PASSERIFORMES	Artamidae	3	3
Sugomel niger	Black Honeyeater	PASSERIFORMES	Meliphagidae	1	3
Syma torotoro	Yellow-billed Kingfisher	CORACIIFORMES	Halcyonidae	1	1
Symposiarchus trivirgatus	Spectacled Monarch	PASSERIFORMES	Monarchidae	1	1
Tachybaptus novaehollandiae	Australasian Grebe	PODICIPEDIFORMES	Podicipedidae	3	3
Tadorna radjah	Radjah Shelduck	ANSERIFORMES	Anatidae	1	1
Tadorna tadornoides	Australian Shelduck	ANSERIFORMES	Anatidae	3	2
Taeniopygia bichenovii	Double-barred Finch	PASSERIFORMES	Estrildidae	2	3
Taeniopygia guttata	Zebra Finch	PASSERIFORMES	Estrildidae	3	3
Tanysiptera sylvia	Buff-breasted Paradise-Kingfisher	CORACIIFORMES	Halcyonidae	3	3
Thalasseus bengalensis	Lesser Crested Tern	CHARADRIIFORMES	Laridae	0	1
Thalasseus bergii	Crested Tern	CHARADRIIFORMES	Laridae	1	3
Thinornis rubricollis	Hooded Plover	CHARADRIIFORMES	Charadriidae	3	3
Threskiornis molucca	Australian White Ibis	CICONIIFORMES	Threskiornithidae	2	2
Threskiornis spinicollis	Straw-necked Ibis	CICONIIFORMES	Threskiornithidae	2	3
Todiramphus chloris	Collared Kingfisher	CORACIIFORMES	Halcyonidae	1	3
Todiramphus macleayii	Forest Kingfisher	CORACIIFORMES	Halcyonidae	3	3
Todiramphus pyrrhopygius	Red-backed Kingfisher	CORACIIFORMES	Halcyonidae	3	3
Todiramphus sanctus	Sacred Kingfisher	CORACIIFORMES	Halcyonidae	3	3
Tregellasia capito	Pale-yellow Robin	PASSERIFORMES	Petroicidae	2	2
Tregellasia leucops	White-faced Robin	PASSERIFORMES	Petroicidae	2	3
Trichoglossus chlorolepidotus	Scaly-breasted Lorikeet	PSITTACIFORMES	Psittacidae	3	2
Trichodere cockerelli	White-streaked Honeyeater	PASSERIFORMES	Meliphagidae	1	2
Trichoglossus haematodus	Rainbow Lorikeet	PSITTACIFORMES	Psittacidae	3	3
Tribonyx mortierii	Tasmanian Native-hen	GRUIFORMES	Rallidae	3	3
Tribonyx ventralis	Black-tailed Native-hen	GRUIFORMES	Rallidae	2	2
Turnix castanotus	Chestnut-backed Button-quail	CHARADRIIFORMES	Turnicidae	3	3
Turnix maculosus	Red-backed Button-quail	CHARADRIIFORMES	Turnicidae	2	2
Turnix melanogaster	Black-breasted Button-quail	CHARADRIIFORMES	Turnicidae	2	1

Turnix olivii	Buff-breasted Button-quail	CHARADRIIFORMES	Turnicidae	3	1
Turnix pyrrhothorax	Red-chested Button-quail	CHARADRIIFORMES	Turnicidae	3	3
Turnix varius	Painted Button-quail	CHARADRIIFORMES	Turnicidae	3	3
Turnix velox	Little Button-quail	CHARADRIIFORMES	Turnicidae	1	2
Tyto javanica	Eastern Barn Owl	STRIGIFORMES	Tytonidae	3	3
Tyto longimembris	Eastern Grass Owl	STRIGIFORMES	Tytonidae	2	1
Tyto novaehollandiae	Masked Owl	STRIGIFORMES	Tytonidae	3	1
Tyto tenebricosa	Sooty Owl	STRIGIFORMES	Tytonidae	3	1
Vanellus miles	Masked Lapwing	CHARADRIIFORMES	Charadriidae	3	3
Vanellus tricolor	Banded Lapwing	CHARADRIIFORMES	Charadriidae	2	3
Xanthotis flaviventer	Tawny-breasted Honeyeater	PASSERIFORMES	Meliphagidae	1	4
Xanthotis macleayanus	Macleay's Honeyeater	PASSERIFORMES	Meliphagidae	2	3
Zoothera heinei	Russet-tailed Thrush	PASSERIFORMES	Turdidae	1	2
Zoothera lunulata	Bassian Thrush	PASSERIFORMES	Turdidae	3	3
Zosterops citrinella	Pale White-eye	PASSERIFORMES	Timaliidae	3	0
Zosterops lateralis	Silvereye	PASSERIFORMES	Timaliidae	3	3
Zosterops luteus	Yellow White-eye	PASSERIFORMES	Timaliidae	1	2

3D grid in visual space

Figure S2. A graphical representation of the process of three-dimensional 'rasterization' of visual space. Each cube in the figure represents a chromatic locus. 'Shadows' on the bottom and side panels are those used in Figs. 1-3 to represent three-dimensional chromatic variation in two dimensions (see text for more details).

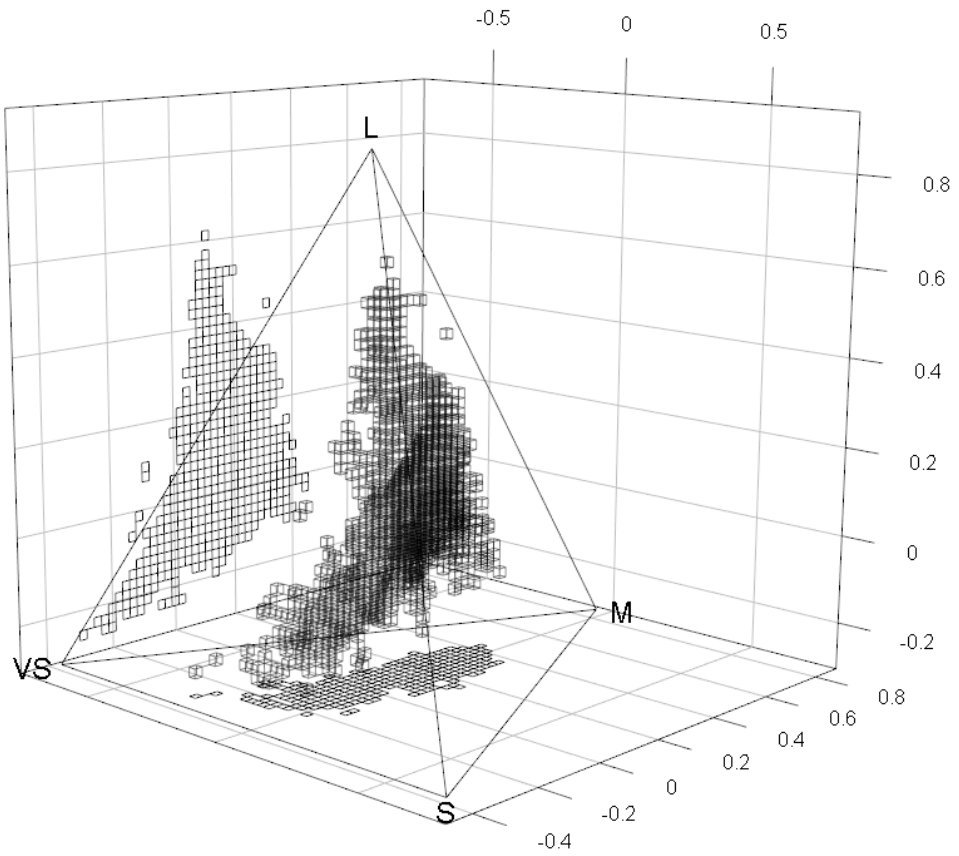

Examples of reflectance spectra

Figure S3. Examples of reflectance spectra classified as melanin-based. Wing scapulars of *Malurus pulcherrimus* male (red), upper back of *Arses telescophthalmus* male (black), upper back *Chroicocephalus novaehollandiae* male (grey), crown *Chroicocephalus novaehollandiae* male (black dashed), belly *Stipiturus malachurus* male (orange) and belly *Malurus melanocephalus* male (thin black).

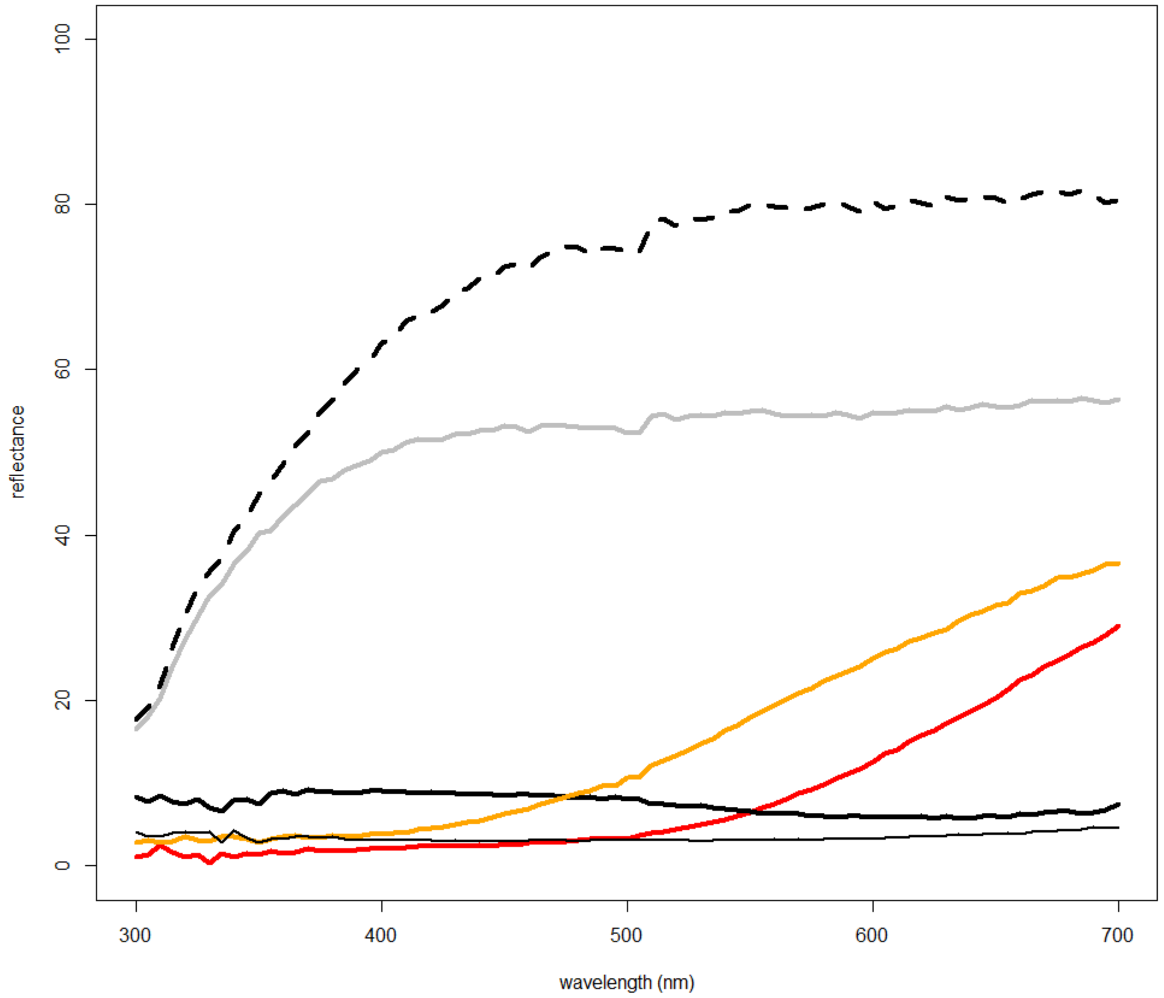

Figure S4. Examples of reflectance spectra classified as carotenoid-based. Cheek of *Stagonopleura oculata* female (red), ventral neck of *Machaerirhynchus flaviventer* (yellow), crown of *Ptilinopus superbus* male (purple), rump of *Acanthiza reguloides* (blue), breast of *Petroica rodinogaster* male (pink), front of *Sericulus chrysocephalus* male (orange).

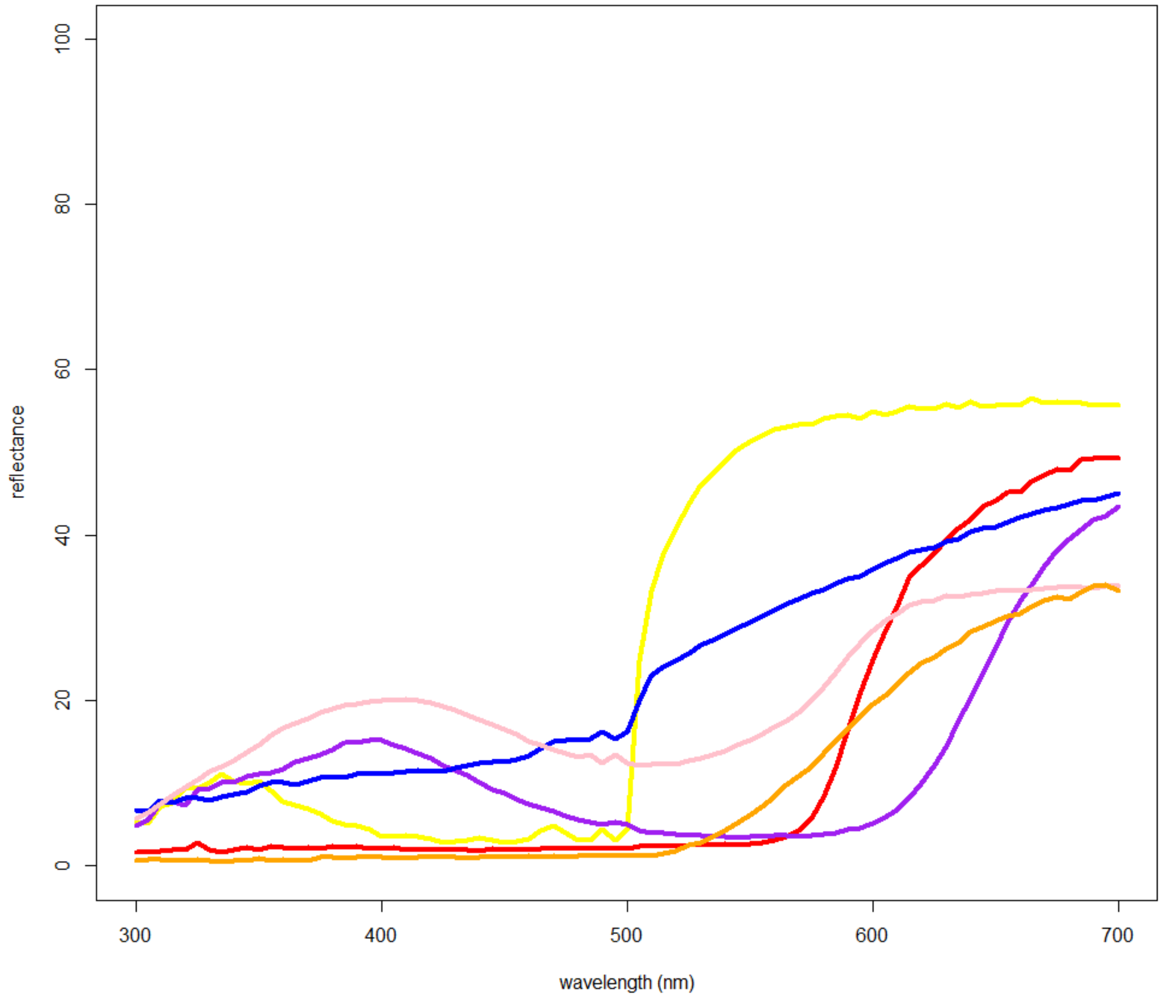

Figure S5. Examples of reflectance spectra classified as psittacofulvin-based. Front of *Platycercus caledonicus* male (red), neck of *Eolophus roseicapillus* (pink), wing scapulars of *Psephotus varius* male (orange), breast of *Neopsephotus bourkii* male (pink dashed), cheek of *Calyptorhynchos funereus* male (yellow) and cheek of *Cacatua galerita* male (yellow dashed).

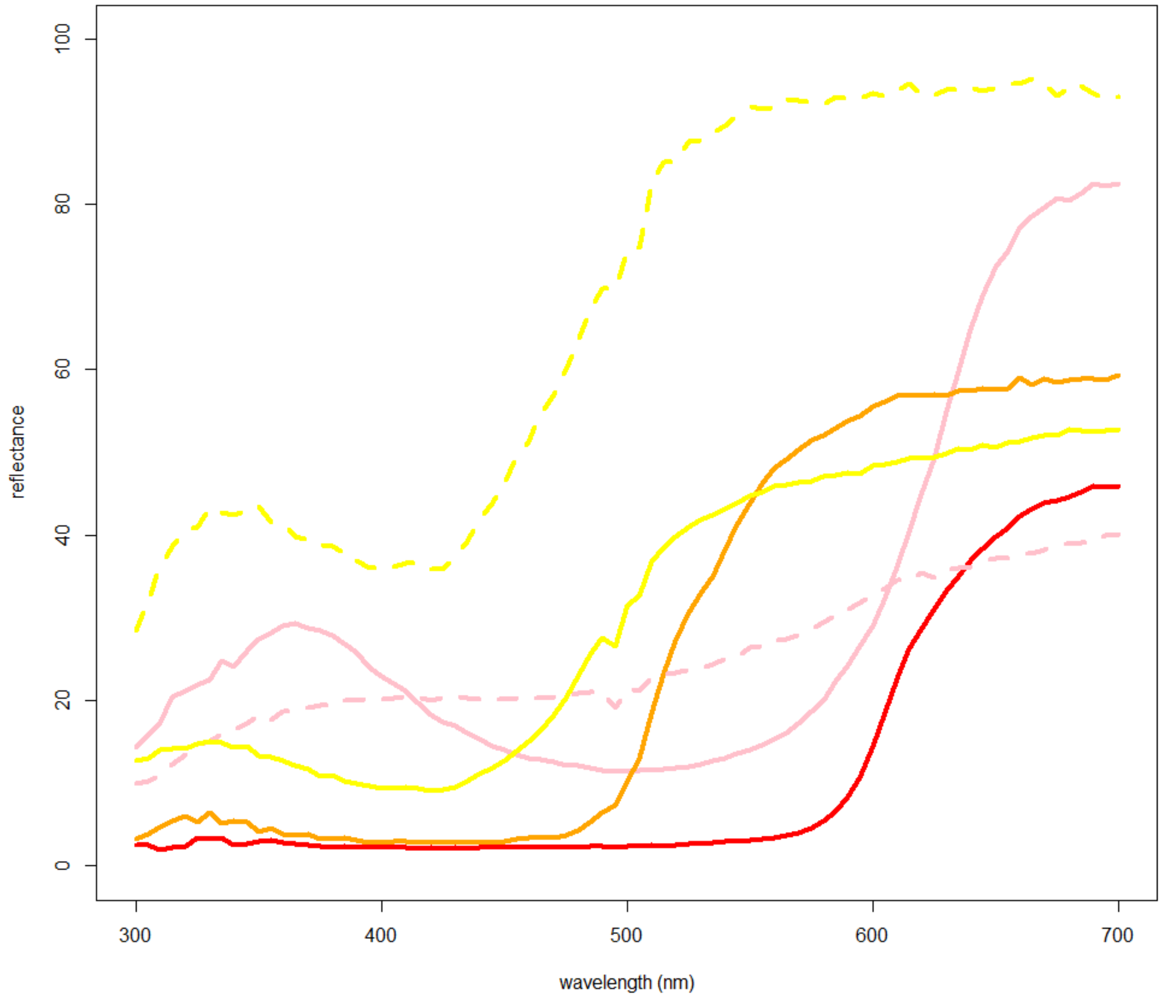

Figure S6. Examples of reflectance spectra classified as structural. Wing scapulars of female *Phaps chalcoptera* (red), upper breast of male *Malurus splendens* (cyan), upper back male *Chalcophaps indica* (green), upper back female *Threskiornis spinicollis* (black), lower back of male *Nettapus pulchellus* (dark green), upper back of male *Aplonis metallica* (black dashed), upper breast male *Erythrura gouldiae* (purple).

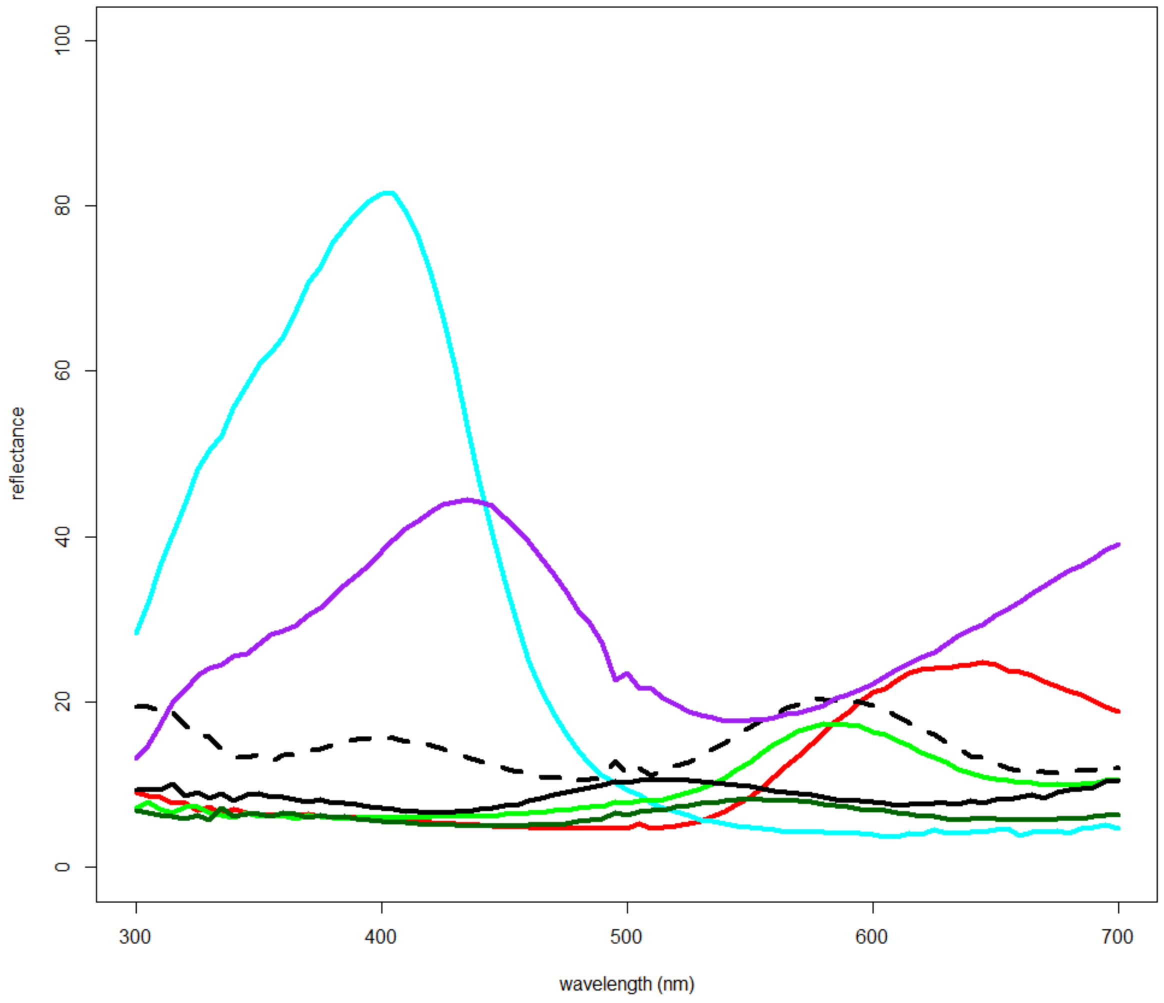

Figure S7. **Examples of reflectance spectra classified as carotenoids+structure.** Ventral neck of *Erythrura trichroa* female (green), rump of *Ailuroedus crassirostris* male (green dashed), upper back of *Ptilinopus superbus* female (dark green) and front of *Merops ornatus* male (black). Note that some subtle aspects of spectral shape (absorption peaks at around 450 nm) cannot be appreciated at this scale for darker spectra.

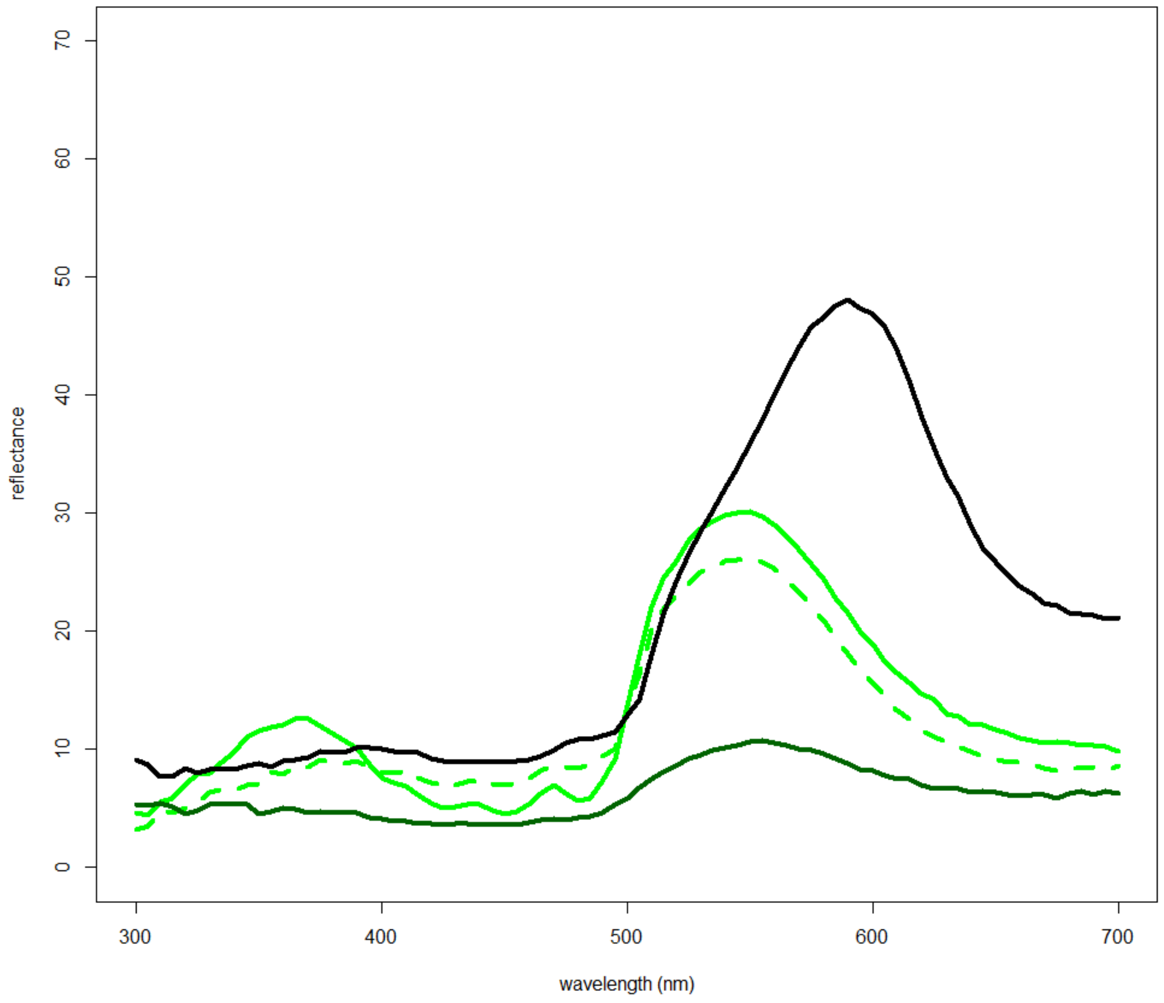

Figure S8. **Examples of reflectance spectra classified as psittacofulvins+structure.** Cheek of male *Aprosmictus erythropterus* (green), lower breast of female *Melopsittacus undulatus* (dark green) and crown of male *Eclectus roratus* (black).

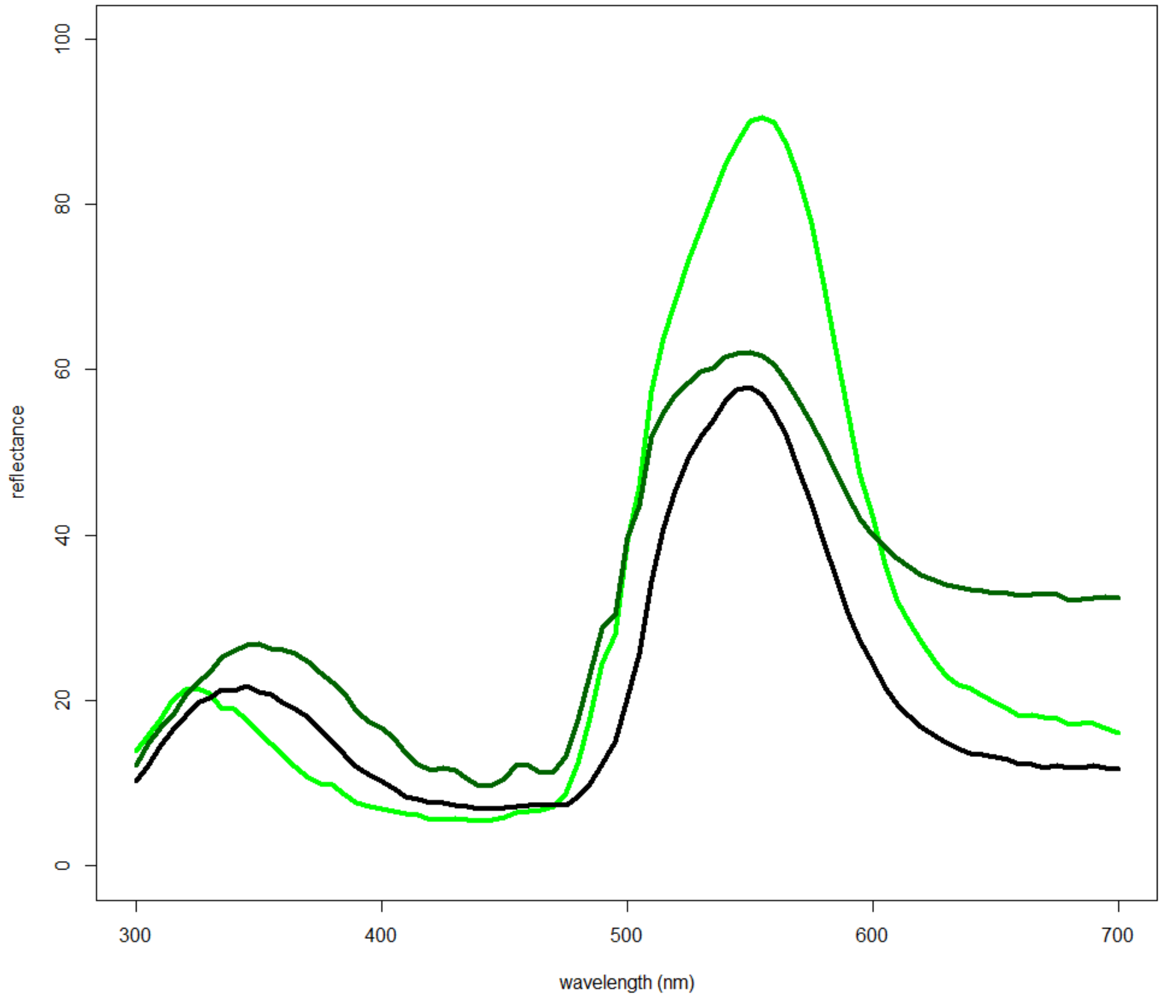

Summary of results for V-type visual systems

The total volume occupied by Australian landbirds modelled using V-type visual sensitivities was 0.055, or 16% of the total theoretical avian colour space. Similar to U-type eyes most colours were concentrated in the centre of the visual space.

Colour production mechanisms

Table S2. Colour space occupation by the main colour-production mechanisms in birds.

	% of total volume	% of total chromatic loci	% of unique chromatic loci
Melanins	4.2	16.3	5.8
Carotenoids	18.8	22.8	10.7
Psittacofulvins	13.3	23.1	11.1
Structural	43.8	39.9	28.5
Carotenoids+structure	2.6	10.3	1.8
Psittacofulvins+structure	18.2	28	16.1

Sex differences

The total volume of chromatic space occupied by males was 0.054 and by females 0.031. This corresponds to 98% and 55.5% of the total chromatic volume of the sample. The overlap in convex hull volume between the sexes comprises 99% of female- and 56% of male-occupied volume. In terms of chromatic loci females occupy 59% and males 79% of all chromatic loci; 39% of occupied loci are shared by the sexes, 21% are restricted to females and 40% to males. Bootstrapping confirms that males occupy larger portions of the chromatic space than females (bootstrapped ratio female/male volume, median = 0.56, 2.5% quantile = 0.39, 97.5% quantile = 0.75; ratio female/male occupied chromatic loci: median = 0.77, 2.5% quantile = 0.73, 97.5% quantile = 0.81).

Taxonomic differences

The orders Passeriformes and Psittaciformes occupy the largest proportion of the colours space (>70% of volume and > 50% of chromatic loci). However, only Psittaciformes show higher values than expected for their number of species (Supplementary Tables S3 and S4). Most orders show expected or lower than expected levels of occupation of the visual space. The only exception was

the order Coraciiformes (>10% of colour volume and >14% of chromatic loci) which had higher levels than expected (see Supplementary Tables S3 and S4).

Table S3. **Comparing observed values of male colour diversity (computed for V-type visual system) for each bird order** (% occupied convex hull volume and chromatic loci) with the expected values obtained from bootstrapping the same number of random species from the entire sample of male colours. P-values are two-tailed and represent the proportion of bootstrapped values more extreme than the observed value.

			% of male convex hull volume				% of male occupied chromatic loci			
	ORDER	n	observed	2.5% quantile bootstrap	97.5% quantile bootstrap	p-value	observed	2.5% quantile bootstrap	97.5% quantile bootstrap	p-value
1	ACCIPITRIFORMES	18	0.005	0.018	0.398	<0.001	0.057	0.068	0.147	0.001
2	ANSERIFORMES	18	0.030	0.018	0.398	0.033	0.081	0.068	0.147	0.062
3	APODIFORMES	2	<0.001	<0.001	0.107	0.125	0.016	0.012	0.034	0.087
4	CAPRIMULGIFORMES	6	<0.001	0.001	0.207	<0.001	0.028	0.031	0.075	0.006
5	CASUARIIFORMES	1	<0.001	<0.001	0.052	0.003	0.004	0.005	0.020	0.003
6	CHARADRIIFORMES	35	0.003	0.073	0.546	<0.001	0.066	0.112	0.215	<0.001
7	CICONIIFORMES	35	0.012	0.014	0.378	0.009	0.070	0.064	0.136	0.035
8	COLUMBIFORMES	22	0.077	0.030	0.441	0.080	0.110	0.079	0.165	0.179
9	CORACIIFORMES	12	0.109	0.008	0.321	0.192	0.141	0.051	0.116	0.001
10	CUCULIFORMES	10	0.009	0.005	0.286	0.041	0.051	0.044	0.103	0.050
11	FALCONIFORMES	6	0.001	0.001	0.207	0.001	0.034	0.031	0.075	0.039
12	GALLIFORMES	6	0.001	0.001	0.207	0.008	0.029	0.031	0.075	0.009
13	GRUIFORMES	16	0.028	0.014	0.378	0.042	0.065	0.064	0.136	0.017
14	PASSERIFORMES	305	0.738	0.533	0.938	0.234	0.509	0.509	0.670	0.013
15	PHALACROCORACIFORMES	6	0.001	0.001	0.207	0.004	0.023	0.031	0.075	0.001
16	PODICIPEDIFORMES	3	0.001	0.000	0.132	0.057	0.016	0.017	0.046	0.007
17	PSITTACIFORMES	49	0.730	0.133	0.630	0.002	0.529	0.144	0.263	0.000
18	STRIGIFORMES	8	0.001	0.003	0.257	0.002	0.040	0.038	0.089	0.025

Table S4. **Comparing observed values of female colour diversity (computed for V-type visual system) for each bird order** (% occupied convex hull volume and chromatic loci) with the expected values obtained from bootstrapping the same number of random species from the entire sample of female colours. P-values are two-tailed and represent the proportion of bootstrapped values more extreme than the observed value.

			% of female convex hull volume				% of female occupied chromatic loci			
	Order	n	observed	2.5% quantile bootstrap	97.5% quantile bootstrap	p-value	observed	2.5% quantile bootstrap	97.5% quantile bootstrap	p-value
1	ACCIPITRIFORMES	18	0.006	0.019	0.379	<0.001	0.078	0.087	0.178	0.004
2	ANSERIFORMES	18	0.050	0.019	0.379	0.098	0.087	0.087	0.178	0.014
3	APODIFORMES	2	0.001	<0.001	0.086	0.097	0.023	0.016	0.044	0.123
4	CAPRIMULGIFORMES	6	0.001	0.002	0.201	0.002	0.039	0.041	0.095	0.010
5	CASUARIIFORMES	1	<0.001	<0.001	0.032	0.003	0.005	0.008	0.025	0.005
6	CHARADRIIFORMES	35	0.009	0.062	0.546	<0.001	0.111	0.134	0.258	0.001
7	CICONIIFORMES	35	0.025	0.016	0.356	0.042	0.083	0.080	0.167	0.023
8	COLUMBIFORMES	22	0.081	0.026	0.414	0.126	0.144	0.098	0.201	0.254
9	CORACIIFORMES	12	0.153	0.009	0.293	0.091	0.163	0.065	0.144	0.002
10	CUCULIFORMES	10	0.013	0.006	0.256	0.056	0.057	0.057	0.129	0.013
11	FALCONIFORMES	6	0.002	0.002	0.201	0.012	0.052	0.041	0.095	0.114
12	GALLIFORMES	6	0.001	0.002	0.201	0.001	0.041	0.041	0.095	0.016
13	GRUIFORMES	16	0.034	0.016	0.356	0.069	0.087	0.080	0.167	0.034
14	PASSERIFORMES	305	0.347	0.507	0.942	<0.001	0.456	0.536	0.706	<0.001
15	PHALACROCORACIFORMES	6	0.001	0.002	0.201	0.001	0.028	0.041	0.095	<0.001
16	PODICIPEDIFORMES	3	0.001	<0.001	0.130	0.032	0.021	0.025	0.059	0.006
17	PSITTACIFORMES	49	0.827	0.100	0.624	<0.001	0.609	0.168	0.307	<0.001
18	STRIGIFORMES	8	0.001	0.004	0.243	<0.001	0.042	0.051	0.114	0.002
